# Tomato (*Solanum lycopersicum* L.) SlIPT3 and SlIPT4 isopentenyltransferases mediate salt stress response in tomato

**DOI:** 10.1186/s12870-015-0415-7

**Published:** 2015-03-12

**Authors:** Eva Žižková, Petre I Dobrev, Yordan Muhovski, Petr Hošek, Klára Hoyerová, Daniel Haisel, Dagmar Procházková, Stanley Lutts, Václav Motyka, Imène Hichri

**Affiliations:** Institute of Experimental Botany, Academy of Sciences of the Czech Republic, Prague, 165 02 Czech Republic; Département Sciences du vivant, Centre wallon de Recherches Agronomiques, Gembloux, B-5030 Belgium; Department of Biomedical Informatics, Faculty of Biomedical Engineering, Czech Technical University in Prague, Kladno, 272 01 Czech Republic; Groupe de Recherche en Physiologie Végétale (GRPV), Earth and Life Institute - Agronomy (ELI-A), Université catholique de Louvain (UCL), Louvain-la-Neuve, 1348 Belgium; Institut National de la Recherche Agronomique, Institut Sophia Agrobiotech (ISA), UMR INRA 1355, CNRS 7254, Université de Nice-Sophia Antipolis, 400 route des Chappes, BP167, F-06903 Sophia-Antipolis Cedex, France

**Keywords:** Cytokinin, Isopentenyltransferase, Salt stress, SlIPT3, SlIPT4, Tomato

## Abstract

**Background:**

Cytokinins (CKs) are involved in response to various environmental cues, including salinity. It has been previously reported that enhancing CK contents improved salt stress tolerance in tomato. However, the underlying mechanisms of CK metabolism and signaling under salt stress conditions remain to be deciphered.

**Results:**

Two tomato isopentenyltransferases, SlIPT3 and SlIPT4, were characterized in tomato and Arabidopsis. Both proteins displayed isopentenyltransferase (IPT) activity *in vitro*, while their encoding genes exhibited different spatio-temporal expression patterns during tomato plant development. *SlIPT3* and *SlIPT4* were affected by the endogenous CK status, tightly connected with CKs feedback regulation, as revealed by hormonal treatements. In response to salt stress, *SlIPT3* and *SlIPT4* were strongly repressed in tomato roots, and differently affected in young and old leaves. *SlIPT3* overexpression in tomato resulted in high accumulation of different CK metabolites, following modifications of CK biosynthesis-, signaling- and degradation-gene expression. In addition, *35S::SlIPT3* tomato plants displayed improved tolerance to salinity consecutive to photosynthetic pigments and K^+^/Na^+^ ratio retention. Involvement of *SlIPT3* and *SlIPT4* in salt stress response was also observed in Arabidopsis *ipt3* knock-out complemented plants, through maintenance of CK homeostasis.

**Conclusions:**

SlIPT3 and SlIPT4 are functional IPTs encoded by differently expressed genes, distinctively taking part in the salinity response. The substantial participation of *SlIPT3* in CK metabolism during salt stress has been determined in *35S::SlIPT3* tomato transformants, where enhancement of CKs accumulation significantly improved plant tolerance to salinity, underlining the importance of this phytohormone in stress response.

**Electronic supplementary material:**

The online version of this article (doi:10.1186/s12870-015-0415-7) contains supplementary material, which is available to authorized users.

## Background

Tomato (*Solanum lycopersicum* L.) is one of the most popular vegetable species grown world-wide because of its edible fruit and agronomic importance. Nevertheless, its production is frequently threatened by environmental stresses such as drought and salinity. High salt concentration reduces tomato germination, leaf number and area, slows down shoot and root growth and increases root/shoot ratio, induces leaf senescence, and ultimately impairs crop production [1]. Salinity imposes both an osmotic stress due to water shortage, followed by an ionic stress resulting from disproportionate nutrients accumulation and an augmentation of toxic ion concentrations, such as sodium [2,3]. Modifications of plant physiological processes following salinization are partly attributed to alterations in phytohormone metabolism. This may be true especially for cytokinins (CKs). Indeed, under long-term salt stress the level of bioactive CKs in tomato plants was reduced down to 50% both in roots and leaves [2,4,5].

The ability to control cell division and differentiation, root growth, leaf senescence, apical dominance, branching, flower and seed development as well as germination and nutrients uptake into sink organs defines CKs as essential regulatory substances in plants [6]. Chemically, CKs are derivatives of adenine substituted at the *N*^6^ position either with isoprenoid or an aromatic side chain. Both isoprenoid and aromatic CKs are present in plants in bioactive forms, as free bases and corresponding nucleosides and nucleotides, and in non-active or storage forms as conjugates with glucose (CK-*O*- and CK-*N*-glucosides) for instance. The typical representatives of isoprenoid CKs are derivatives of *trans*-zeatin (*t*Z), *cis*-zeatin (*c*Z), dihydrozeatin (DHZ) and *N*^6^-(Δ^2^-isopentenyl)adenine (iP), occuring throughout the plant kingdom [7,8]. The first step of CK biosynthesis is performed by isopentenyltransferases (IPTs). Plant *IPTs* belong to multigenic families, chiefly described in Arabidopsis (*AtIPT1*-*AtIPT9*) [9,10] and in maize, for instance [11]. Recently, *IPT* genes have also been identified in tomato (*SlIPT1-SlIPT6*), and their expression patterns specifically investigated during fruit set and development [12]. IPTs catalyze the transfer of the isoprenoid moiety from precursors dimethylallyl diphosphate (DMAPP) or (*E*)-4-hydroxy-3-methyl-but-2-enyl diphosphate (HMBDP) to adenine nucleotide forms (AMP, ADP, ATP) [9,10]. Modulation of CK contents through modification of *IPT* expression strongly impacts plants development. Indeed, while overexpression of *IPT* in plants resulted in faster shoot formation, shorter internodes, loss of apical dominance, delay of leaf senescence, higher photosynthetic rates and accumulation of *t*Z and its riboside [13-16], Arabidopsis *ipt* deficient plants showed strong inhibition of shoot growth, elongation of primary and lateral roots, and reduction of *t*Z and iP contents [17].

Recent progress in genetic engineering of CKs enabled to control plant CK contents, affected plant traits, increased yield production and improved plant adaptation to enviromental stresses such as salinity [18-20]. Indeed, responsiveness of Arabidopsis *IPT* genes to salt stress has been demonstrated by transcriptome analyses, where upregulation of *AtIPT1*, *AtIPT2* and *AtIPT8* and downregulation of *AtIPT3*, *AtIPT5*, *AtIPT7* and *AtIPT9* were reported [21]. Regulation of *IPT* expression through inducible promoter in tomato roots improved tolerance to salt stress and led to higher yield compared to the wild-type plants [3,4]. Similarly, plant salinity tolerance was enhanced in transgenic cotton expressing *IPT* under control of a *cysteine proteinase* (*Ghcysp*) promoter, delaying the salt-induced senescence of leaves [22]. Positive correlation between *IPT* expression and salt resistance has been demonstrated in transgenic tobacco by introducing *Agrobacterium tumefaciens IPT* under control of the stress-inducible promoter *rd29A* as well [23].

In addition to CK biosynthesis, CK signaling steps are also critical for plant response to salinity. CKs are perceived at the plasma membrane by specific receptors, and the signal is transduced *via* type-B Arabidopsis Response Regulators (ARRs) controlling transcription of type-A ARRs, which act as negative feedback regulators of CK signaling [24]. ARR transcription factors respond in different ways to salt stress and were reported to regulate sodium accumulation in Arabidopsis [25]. Moreover, a transcriptome analysis of Arabidopsis CK deficient *ipt1,3,5,7* mutants exposed to salinity revealed the importance of CK regulation on stress-responsive signaling pathways, even under normal conditions of growth [21].

Although CKs assume crucial functions in tomato salt tolerance, data concerning the underlying molecular cues involved in this process remain unclear. To better understand salt impact on CK metabolism in tomato, a thorough characterization of genes coding for IPTs and their response to salinity is required. The present study reports the functional characterization of two tomato IPT encoding genes, *SlIPT3* and *SlIPT4*, both *in vitro* and *in planta*. Spatio-temporal expression profiles during plant development, enzymatic activity of both proteins and their involvement in CK biosynthesis were determined. Likewise, SlIPT3 and SlIPT4 participation in response to salt stress was investigated in Arabidopsis and tomato.

## Results

### Identification of two putative non-redundant tomato isopentenyltransferases

**Figure 1:**
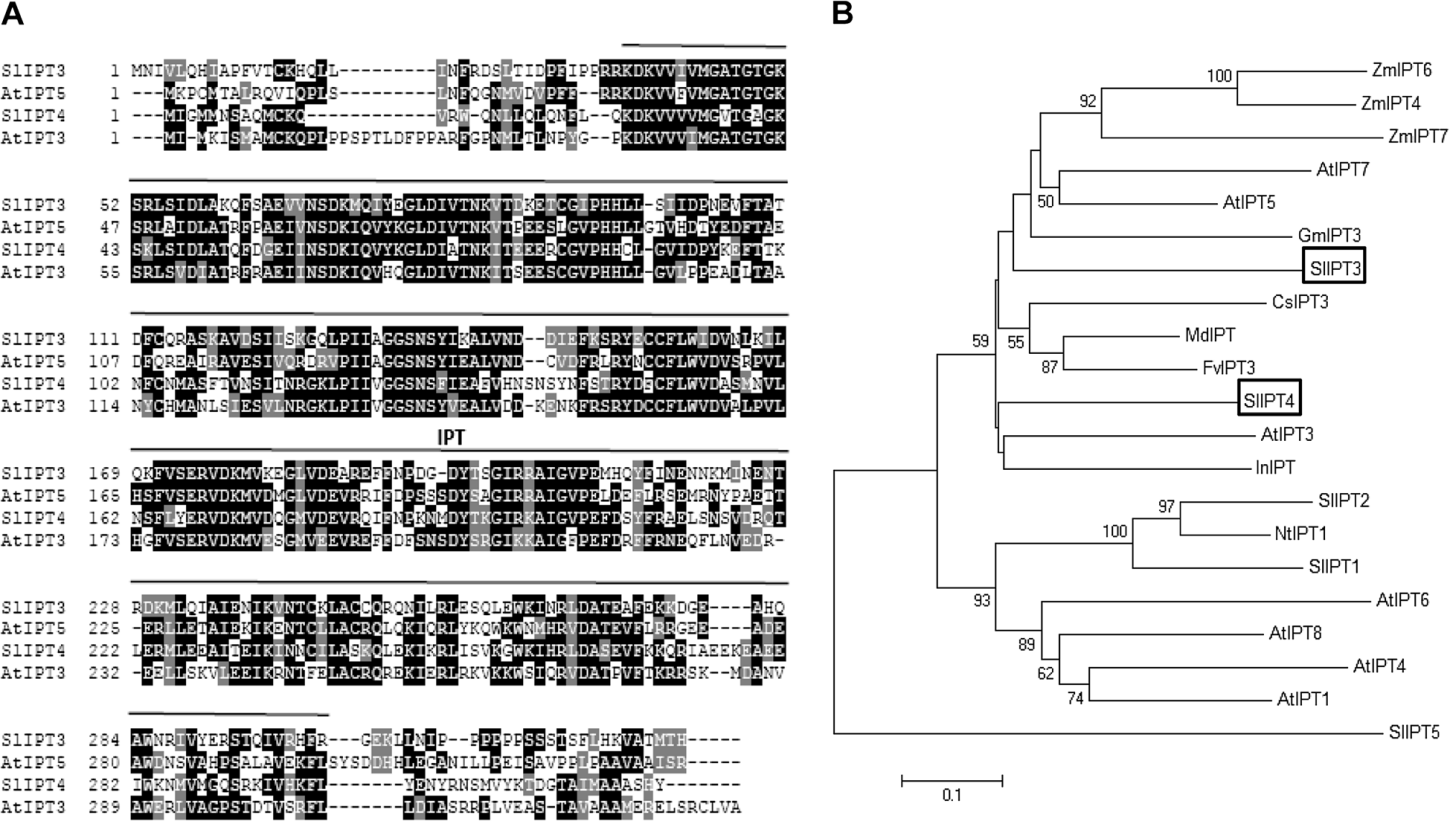
**Identification of SlIPT3 and SlIPT4. (A)** Full-length protein sequence alignment of SlIPT3, SlIPT4 and Arabidopsis AtIPT3 and AtIPT5. Identical residues are colored in black and conserved residues in dark gray. The isopentenyltransferase (IPT) domain is labeled. **(B)** Phylogenetic relationships between SlIPT3 [ADZ28498], SlIPT4 [AEE39459] and related proteins of *Cucumis sativus* CsIPT3 [XP_004136062], *Ipomoea nil* InIPT [BAG55006], *Malus domestica* MdIPT [ADY80558], *Fragaria vesca* FvIPT3 [XP_004290672], *Arabidopsis thaliana* AtIPT3 [Q93WC9], AtIPT4 [Q9SB60], AtIPT5 [Q94ID2], AtIPT6 [Q9C6L1], AtIPT7 [Q94ID1] and AtIPT8 [Q9LJL4], *Glycine max* GmIPT3 [XP_003528670], *Zea mays* ZmIPT4 [ABY78883], ZmIPT6 [ABY78885] and ZmIPT7 [ABY78886], *Solanum lycopersicum* SlIPT1 [BAM08995], SlIPT2 [BAM08996], SlIPT5 [BAM08998] and SlIPT6 [XP_004251888], *Nicotiana tabacum* NtIPT1 [AFV15392]. The phylogenetic tree was constructed according to the neighbor-joining method, using MEGA5 [27]. The percentage of reliability of each branch point of the rooted tree, as assessed by the analysis of 1000 trees (bootstrap replicates), is shown on the branch stem.

To identify IPT encoding genes in tomato, Arabidopsis *IPT1*-*9* coding sequences were used to screen the tomato genome database [26] (www.solgenomics.net). Among the selected expressed sequence tags (ESTs), two were apparently encoding full length proteins and could be amplified by PCR. For the first gene (GenBank accession JF423320), a 1122 bp coding sequence was cloned, including a 990 bp open reading frame (ORF) defining a 329 amino acid (aa) polypeptide of 37.5 kDa (Figure 1A). This gene was recently annotated as *SlIPT3* [12]. For the second gene (GenBank accession JF433930), a 1073 bp coding sequence was amplified, exhibiting a 972 bp ORF predicted to encode a 323 a protein of 37.1 kDa. This gene was classified as *SlIPT4* [12].

*SlIPT3* is located on tomato chromosome 1, while *SlIPT4* is situated on chromosome 9. In addition, *SlIPT3* includes a 293 bp intron localized in the 5′ UTR region. SlIPT3 and SlIPT4 display 52% identity and 72% similarity. Alignment of SlIPT3 and SlIPT4 proteins with Arabidopsis IPT3 and IPT5 indicates that the IPT catalytic domain represents almost the entirety of the proteins, with exception of the N- and C-termini (Figure 1A). A phylogenetic analysis of SlIPT3 and SlIPT4 and nineteen additional orthologs belonging to different species was constructed by means of the neighbour-joining method using full-length amino acid sequences (Figure 1B). It indicated that SlIPT3 mainly clusters with soybean GmIPT3 and Arabidopsis AtIPT5, while SlIPT4 clusters with Arabidopsis AtIPT3 and Japanese Morning Glory InIPT. Except from Arabidopsis and maize, full characterization of IPT enzymes in other plant species is missing.

### Tomato *SlIPT3* and *SlIPT4* show isopentenyltransferase activity *in vitro*

**Figure 2:**
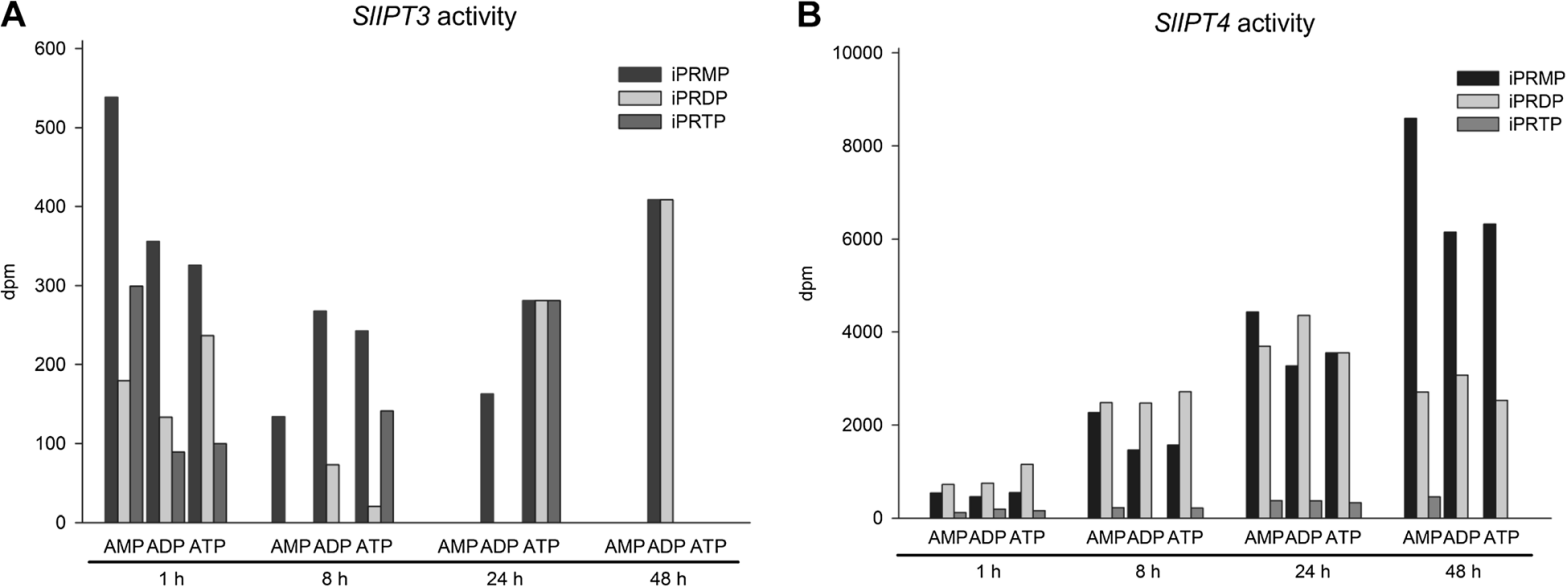
***In vitro*** **determination of DMAPP:AMP, ADP and ATP isopentenyltransferase activity of SlIPT3 (A) and SlIPT4 (B) proteins.** ADP: adenosine diphosphate, ATP: adenosine triphosphate, iPMP: isopentenyladenosine-5′monophosphate; iPDP: isopentenyladenosine-5′-diphosphate, iPTP: isopentenyladenosine-5′-triphosphate. Note that vertical scales are not the same for SlIPT3 and SlIPT4.

To examine the ability of SlIPT3 and SlIPT4 to catalyze CK biosynthesis, a functional analysis of both proteins was carried out. As SlIPT3 and SlIPT4 are highly insoluble when produced in bacteria, both proteins were produced *in vitro*. The enzymatic activity of SlIPT3 and SlIPT4 was determined using *in vitro* assays based on conversion of radiolabeled adenylated substrates ([^3^H]AMP, [^3^H]ADP or [^3^H]ATP) in the presence of DMAPP to isopentenylated products. Both enzymes were able to convert tritium-labeled AMP/ADP/ATP to the corresponding iPRMP/iPRDP/iPRTP in time-dependent manner (Figure 2A and B). The prevailing metabolites formed by both SlIPT3 and SlIPT4 were iPRMP and iPRDP, with almost 10-fold higher catalytic activity shown by SlIPT4 compared to SlIPT3. These results clearly demonstrate that SlIPT3 and SlIPT4 display CK biosynthetic activity in *in vitro* conditions.

### Subcellular localization of SlIPT3 and SlIPT4

**Figure 3:**
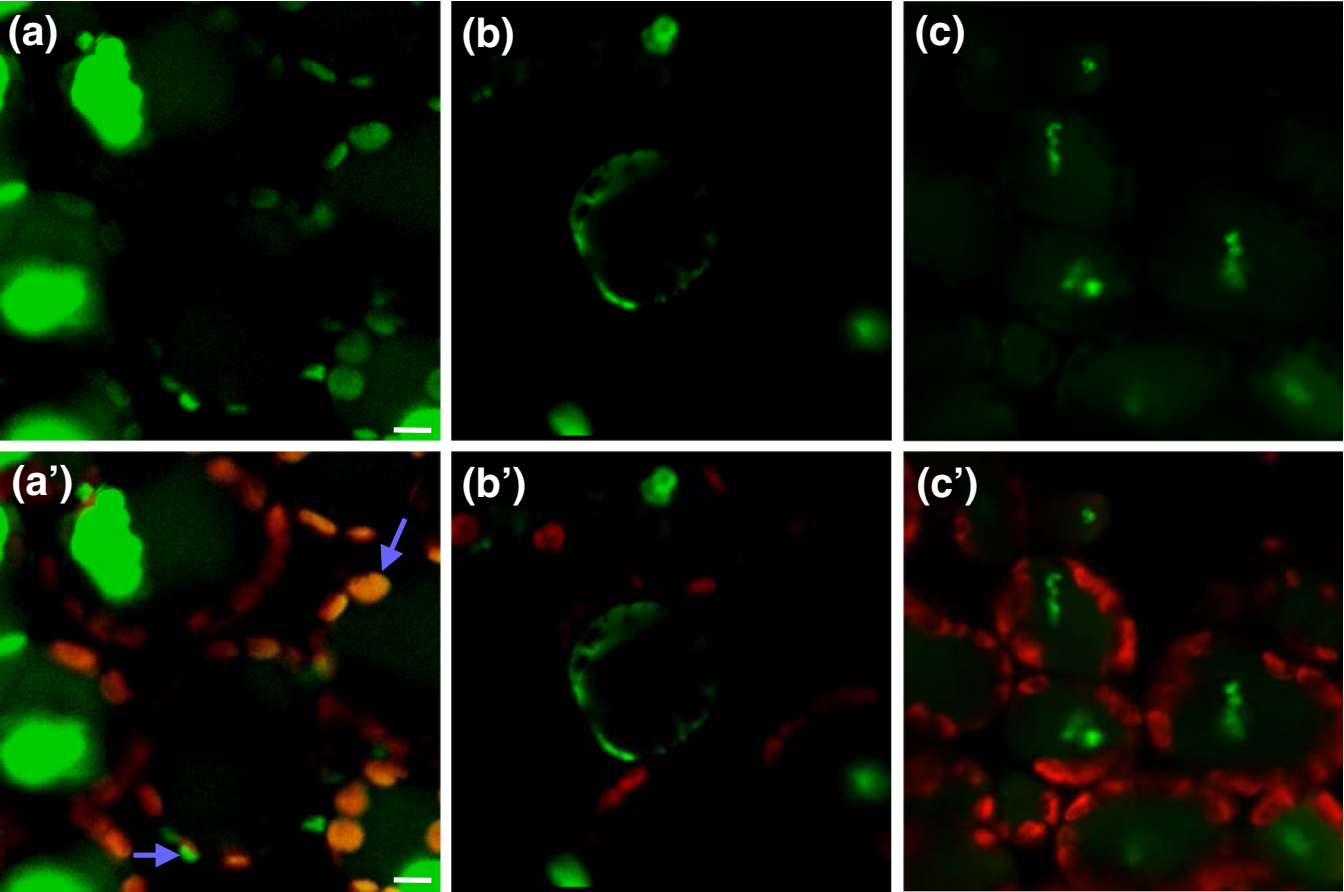
**Subcellular localization of SlIPT4-GFP by tomato leaves agroinfiltration. (a, a‘)** SlIPT4-GFP fluorescence and chlorophyll/GFP fluorescence, respectively. **(b, b‘)** Control GFP fluorescence and chlorophyll/GFP fluorescence, respectively. **(c, c‘)** Control untransformed tomato leaves and chlorophyll autofluorescence, respectively. *Bar* = 4 µm.

An analysis of the deduced SlIPT3 and SlIPT4 sequences revealed the presence of chloroplast transit peptides [28] (pSORT, http://psort.ims.u-tokyo.ac.jp/). To investigate protein subcellular localization, *SlIPT3* and *SlIPT4* cDNA were fused at their C-terminal end to green fluorescent protein (GFP) coding sequence, placed under cauliflower mosaic virus *35S* promoter and used to agro-infiltrate tomato leaves. As observed in Figure 3 (a, a‘), despite a strong auto-fluorescence, SlIPT4 showed localization predominantly in chloroplasts and in the cytosolic area surrounding the chloroplast. Indeed, GFP fluorescence colocalized with chlorophyll fluorescence in this cellular compartment. In contrast, free GFP was restricted to the cytoplasm (Figure 3 (b, b‘)), while no fluorescence at 520 nm (GFP emission wavelength) was detected in untransformed tomato leaves (Figure 3 (c, c‘)). Despite several attempts, we could not establish subcellular localization for SlIPT3, and no GFP signal of the recombinant protein could be detected in transformed tomato cells. These data show that at least SlIPT4 is localized in the chloroplast and in the cytosolic area surrounding chloroplasts.

### Characterization of SlIPT3 and SlIPT4 in Arabidopsis *ipt3* mutants

In parallel, tomato *SlIPT3* and *SlIPT4* were functionally characterized *in planta via* complementation of Arabidopsis *ipt3* knock-out (*ipt3* KO) mutants. Five days after sowing (DAS), the Arabidopsis wild-type (WT) plants, *ipt3* KO mutants and Arabidopsis *ipt3* KO complemented plants showed a similar phenotype on half-strength MS medium (Additional file 1). We thus suggested to determine the function of SlIPT3 and SlIPT4 in Arabidopsis complemented *ipt3* KO mutants grown on salt medium and to follow their response to salinity. Based on the highest phenotype discrimination, 100 mM NaCl medium was used for further testing of seed germination and plant survival percentages, primary root elongation, seedlings CKs content and CK-related genes expression profiles.

**Figure 4:**
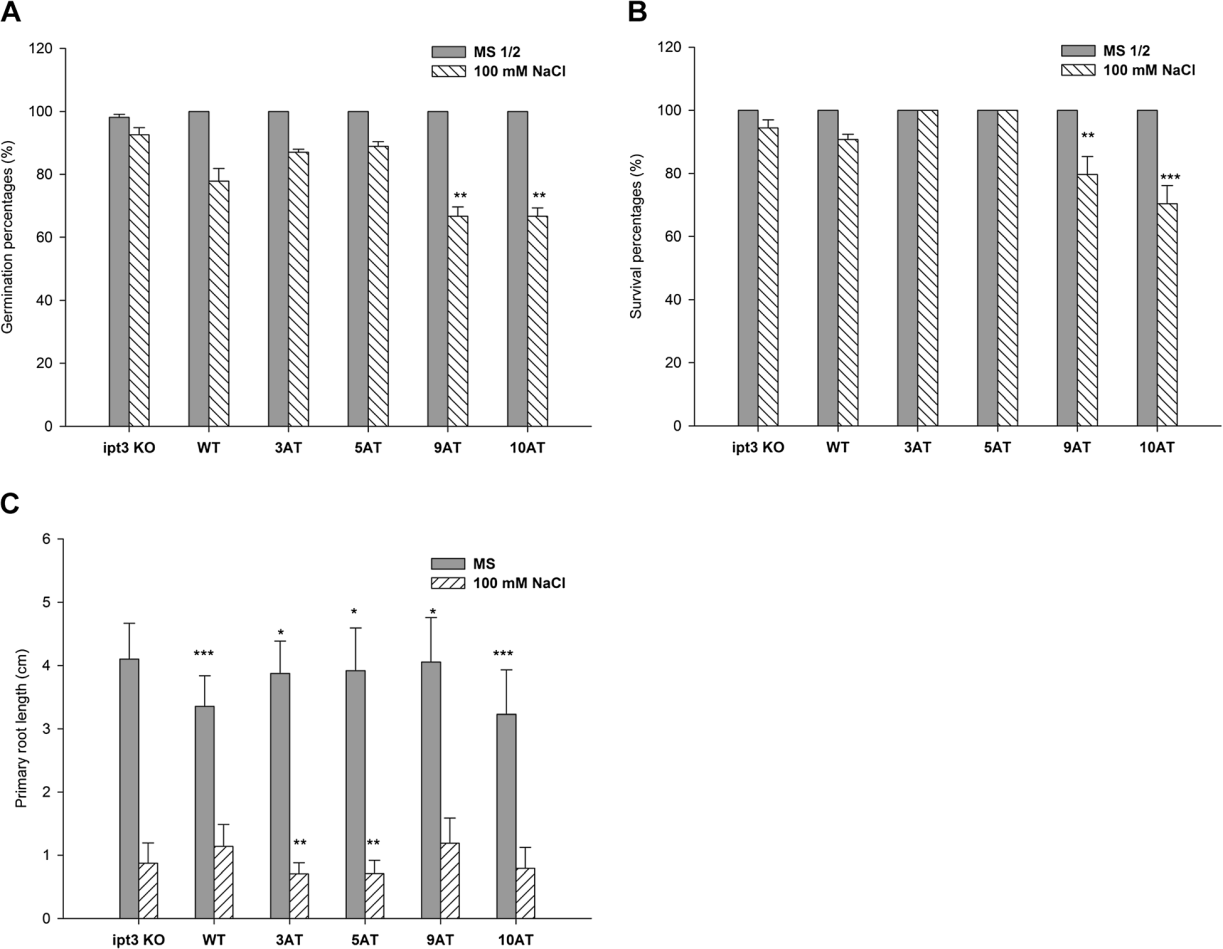
**Salinity response of SlIPT3 and SlIPT4 in Arabidopsis.** Germination percentage **(A)** and survival percentage **(B)** of *SlIPT4* (lines 3AT and 5AT) and *SlIPT3* (lines 9AT and 10AT) Arabidopsis *ipt3* complemented plants. **significantly lower germination and survival percentages compared to *ipt3* KO. ***significantly lower survival percentages compared to WT and *ipt3* KO. **(C)** Primary root length of Arabidopsis *SlIPT3* and *SlIPT4* complemented plants. *significantly longer primary root compared to WT. **significantly shorter primary root compared to WT. ***significantly shorter primary root compared to *ipt3* KO plants (Mann–Whitney U Test with p ≤ 0.05).

On the control medium, germination and survival frequencies were not significantly different in between the variants reaching 100%. On the saline medium, seed germination percentage was reduced to 67% for both lines of Arabidopsis *SlIPT3* complemented plants (9AT and 10AT lines), significantly less than that of *ipt3* KO and WT plants (Figure 4A). However, in Arabidopsis *SlIPT4* complemented plants (3AT and 5AT lines), seed germination reached 89% and 90%, respectively, while WT seeds displayed 78% and *ipt3* KO 93% of germination rate, respectively. The same trend was detected for seedlings’ survival percentage on 100 mM NaCl (Figure 4B) (Fisher’s exact test with p ≤ 0.05). Similarly, primary root elongation was estimated by screening 10 DAS Arabidopsis *SlIPT3* and *SlIPT4* complemented plants (Figure 4C). On control medium, the primary root length among the 9AT and 10AT lines significantly differed compared to WT while on the salt-containing medium, no significant changes between the *SlIPT3* complemented and WT plants were observed. Arabidopsis plants complemented with *SlIPT4* showed significantly longer primary root (3.77 ± 0.41 and 3.85 ± 0.8 cm for 3AT and 5AT, respectively) than WT (3.35 ± 0.58 cm) on control medium, while antagonistic effects were detected on salt-containing medium (Figure 4C, Mann–Whitney U Test with p ≤ 0.05). These results demonstrate that both tomato IPT enzymes are functional in heterologous system. However, in reaction to salt stress the *SlIPT4* complemented plants displayed the same characteristics as WT in germination and survival assays, while both of the *SlIPT3* complemented lines differed in their response to salinity.

**Figure 5:**
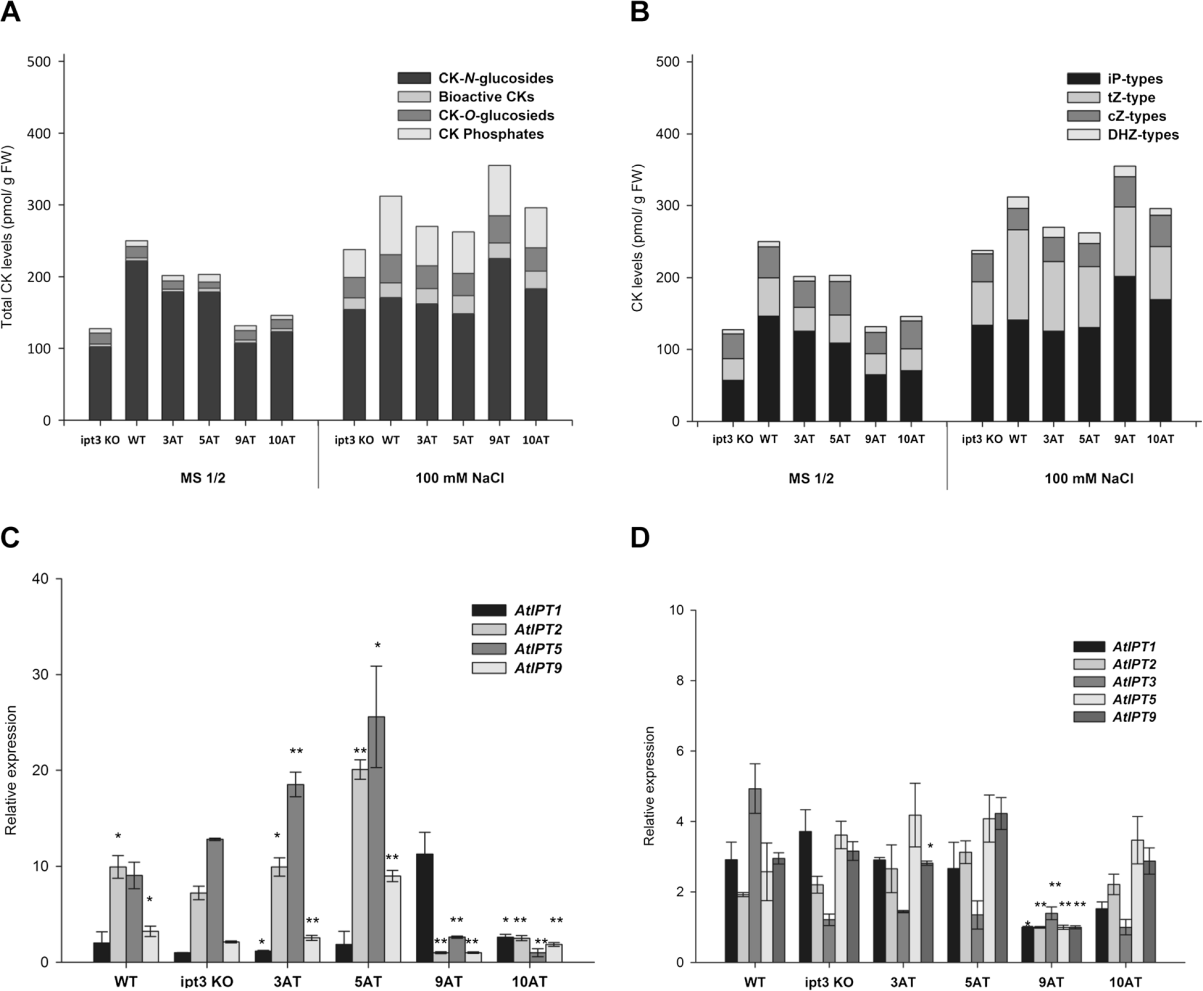
**CKs accumulation in Arabidopsis** ***ipt3*** **complemented plants.** Total endogenous CK levels of *SlIPT4* (lines 3AT and 5AT) and *SlIPT3* (lines 9AT and 10AT) *ipt3* KO complemented plants divided according to the chemical structure **(A)** and contents of bioactive CKs **(B)**. Relative expression of CK biosynthetic genes in Arabidopsis *ipt3* KO mutants complemented plants cultivated on the control medium **(C)** and on the salt-containing (100 mM NaCl) medium **(D)**. *Actin* and *EF1* were used as internal controls for normalization of *AtIPT* transcript levels. Data represent means and SD of two replicates. *statistically significant difference from *ipt3* KO (unpaired two-tailed Student’s *t*-test, p ≤ 0.05). **statistically significant difference from *ipt3* KO after Šidák correction for testing of multiple lines (multiple unpaired two-tailed Student’s *t*-test, overall α = 0.05, individual p ≤ 0.01).

To assess whether salt treatment affected CK metabolism in Arabidopsis *SlIPT3* and *SlIPT4* complemented plants, CK contents and gene expression profiles of CK biosynthetic genes were investigated on control and salt medium (100 mM NaCl). Generally, in both types of media, the total content of CKs of Arabidopsis *SlIPT3* and *SlIPT4* complemented plants exceeded that of *ipt3* KO plants. However, in comparison to WT plants, Arabidopsis *ipt3* complemented plants showed lower CK concentrations in transgenic lines with exception of the 9AT line on salt medium (Figure 5A). Proportionally, CK-*N*-glucosides accumulated strongly in all tested variants (149.01 – 254.81 pmol/g FW), with iP7G as the predominant metabolite regardless of growth medium (Additional file 2 and Additional file 3). Salt treatment substantially increased the levels of CK free bases and ribosides, while *t*Z-type CKs were mainly enhanced among the CK groups (Figure 5B).

The expression profiles of CK biosynthetic genes were investigated in Arabidopsis seedlings. No expression in vegetative phase of development was detected for *AtIPT4*, *AtIPT6*, and *AtIPT8*, as had been previously observed [17]. On the control medium, complemented plants showed differential expression of the remaining *IPT* genes compared to WT or *ipt3* KO plants. *AtIPT2* and *AtIPT5* were repressed in Arabidopsis *SlIPT3* complemented plants, while their expression increased in Arabidopsis *SlIPT4* complemented plants, in comparison with WT or *ipt3* KO plants (Figure 5C). Salinity strongly downregulated *AtIPT2* and *AtIPT5* expression, while it increased expression of *AtIPT3* in WT, *ipt3* KO and *SlIPT4* complemented plants (Figure 5D). In *SlIPT3* complemented plants, genes were differentially regulated depending on the transgenic line considered (9AT or 10AT). These results demonstrate that salinity differently affects *IPT* genes expression and CK status in Arabidopsis, and that SlIPT3 and SlIPT4 show distinct regulatory mechanisms in heterologous system. Therefore we propose that Arabidopsis plants respond to salt stress by the elevation of CK levels, with predominant accumulation of *N*-glucosides suggesting overabundance of CKs that is followed by downregulation of some IPT encoding genes to maintain CK homeostasis.

### *SlIPT3* and *SlIPT4* are differently expressed in tomato organs and participate in cytokinin homeostasis

**Figure 6:**
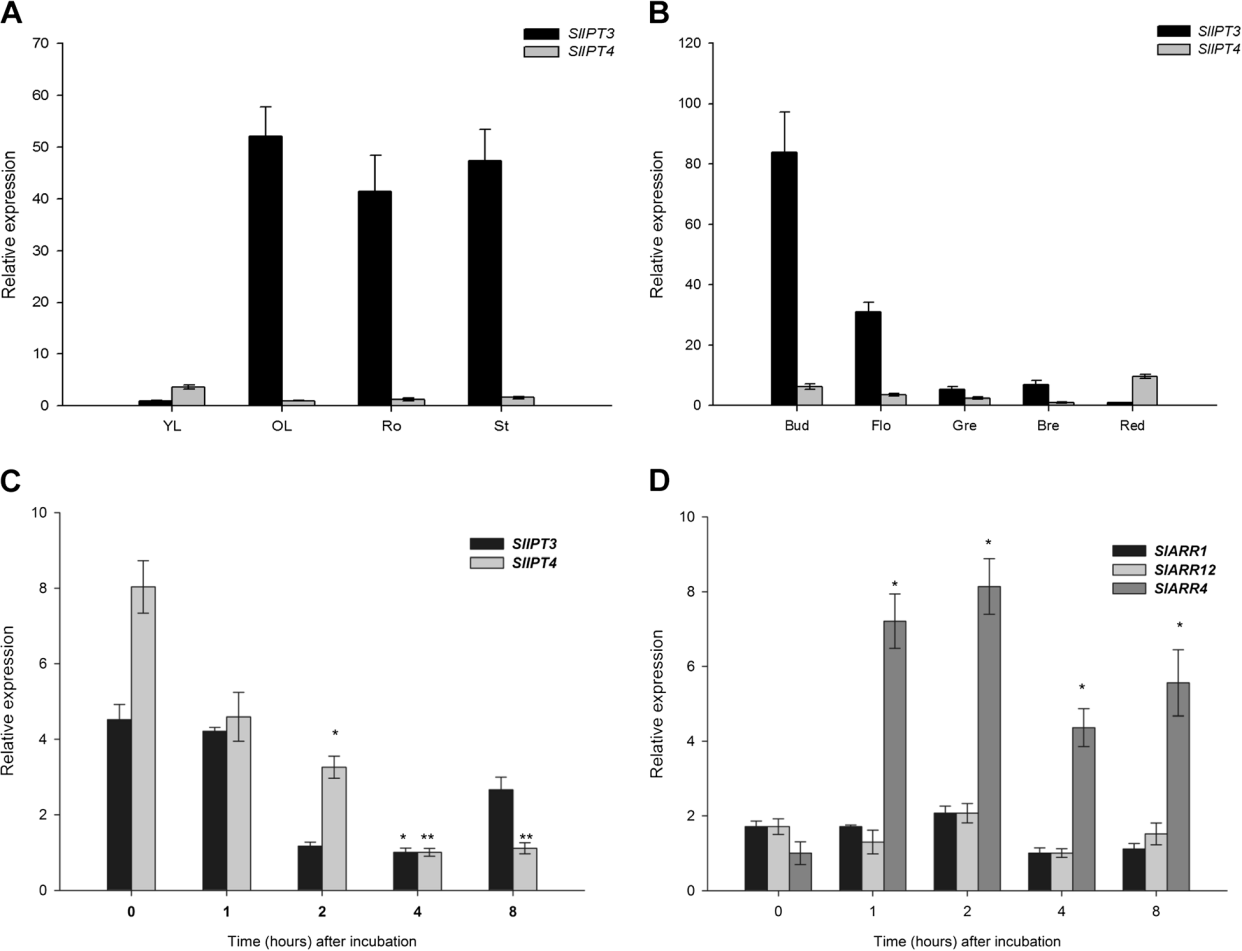
**QRT-PCR analysis of** ***SlIPT3*** **and** ***SlIPT4*** **expression.** Spatio-temporal expression profile during tomato plant development in vegetative organs **(A)** and reproductive organs **(B)**. Regulation of *SlIPT3* and *SlIPT4* expression by CK treatment: Effects of *t*Z (10 μM) treatment on *SlIPT3* and *SlIPT4* expression **(C)** and the expression levels of CK response regulators *SlARR1*, *SlARR4* and *SlARR12* **(D)**.* Actin* and *GAPDH* were used as internal controls for normalization of *SlIPT3* and *SlIPT4* transcript levels. Data represent means and SD of three replicates. *statistically significant difference from time 0 h (unpaired two-tailed Student’s *t*-test, p ≤ 0.05). **statistically significant difference from time 0 h after Šidák correction for testing of multiple points (multiple unpaired two-tailed Student’s *t*-test, overall α = 0.05, individual p ≤ 0.0127). YL, young leaves; OL, old leaves; Ro, roots; St, stem; Bud, bud; Flo, flower; Gre, green stage; Bre, breaker stage; Red, red stage of tomato development.

The expression patterns of *SlIPT3* and *SlIPT4* in various tomato vegetative and reproductive organs from plants cultivated in the greenhouse under normal growth conditions, including three stages of fruit development and ripening, were investigated by qRT-PCR (Figure 6A and B). In general, *SlIPT3* transcripts were much more abundant than *SlIPT4* ones in all the tested organs. Both genes were expressed in young and old leaves, roots and stems of tomato plants. In vegetative tissues, *SlIPT3* transcripts were weakly detected in young leaves but highly accumulated in stems, roots and old leaves. In contrast, *SlIPT4* was preferentially expressed in young leaves (Figure 6A). In reproductive tissues, *SlIPT3* transcripts were abundant in buds and flowers, then rapidly decreased during tomato fruit maturation with higher levels of transcripts in the breaker stage. *SlIPT4* was preferentially expressed in buds, then continuously declined until the breaker stage and reached its peak expression at the red stage of tomato fruit (Figure 6B). Taken together, these data indicate that *SlIPT3* and *SlIPT4* display distinct expression profiles during tomato development.

In order to clarify a putative feedback regulation by CKs on *SlIPT3* and *SlIPT4* expression, WT tomato roots from hydroponic plants were treated with *t*Z. CK treatment gradually repressed *SlIPT3* (up to 4-fold) and *SlIPT4* (up to 8-fold) expression, the inhibitory effect being most noticeable 4 h after treatment onset. However, *SlIPT3* transcripts then started to accumulate again, which was not the case for *SlIPT4* (Figure 6C). Likewise, the exogenous application of *t*Z strongly induced expression of the CK negative-feedback ARR type-A *SlARR4*, in contrast to its more discrete effect on ARR type-B *SlARR1* and *SlARR12* (Figure 6D). Altogether, these data suggest a regulation of *SlIPT3* and *SlIPT4* transcripts accumulation through plant endogenous CK status, in addition to the feedback regulation *via* the CK signaling pathway.

### Overexpression of *SlIPT3* strongly impacts tomato phenotype and cytokinin status

**Figure 7:**
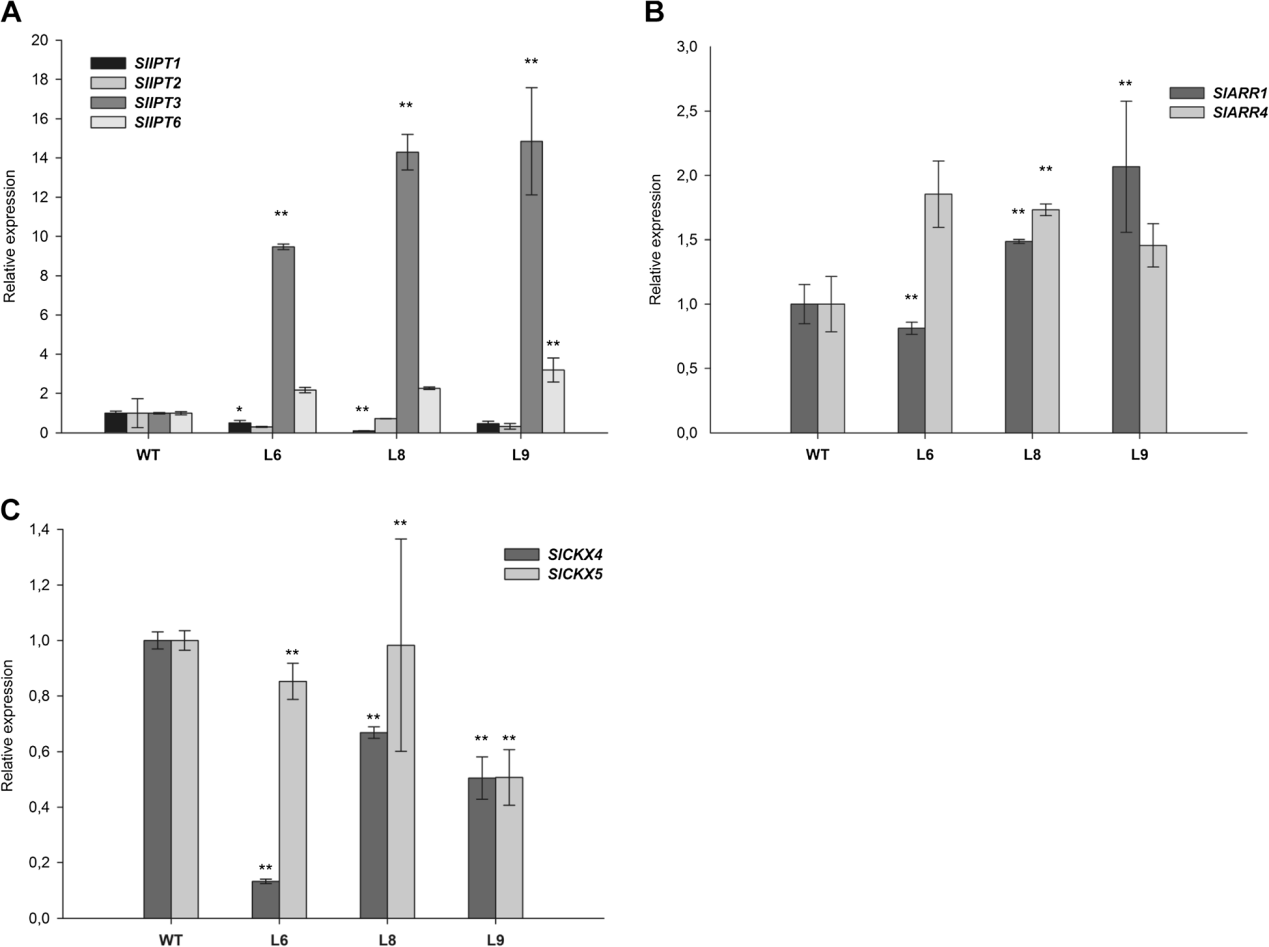
**Relative expression of CK metabolic and response genes in tomato young leaves of** ***35S::SlIPT3*** **and WT plants.** Transcript abundance of CK biosynthetic genes **(A)**, genes involved in CK signaling **(B)** and degradation **(C)** pathways. *Actin* and *GAPDH* were used as internal controls for normalization of candidate genes expression. Data represent means and SD of two replicates. *statistically significant difference from WT (unpaired two-tailed Student’s *t*-test, p ≤ 0.05). **statistically significant difference from WT after Šidák correction for testing of multiple lines (multiple unpaired two-tailed Student’s t-test, overall α = 0.05, individual p ≤ 0.017).

To understand the impact of *SlIPT3* and *SlIPT4* overexpression on CK metabolism and verify the enzymes activity *in planta*, we attempted to generate transgenic lines constitutively expressing *SlIPT3* or *SlIPT4*. Several *35S*::*SlIPT3* transgenic lines were able to develop and survive repotting into soil, while no *35S*::*SlIPT4* tomato plants could be regenerated from transgenic calli *in vitro*. For further characterization, three *35S*::*SlIPT3* transgenic lines (L6, L8, L9) were selected, with transgene expression in leaves increasing up to 14-fold (L9) compared to WT (Figure 7A).

**Figure 8:**
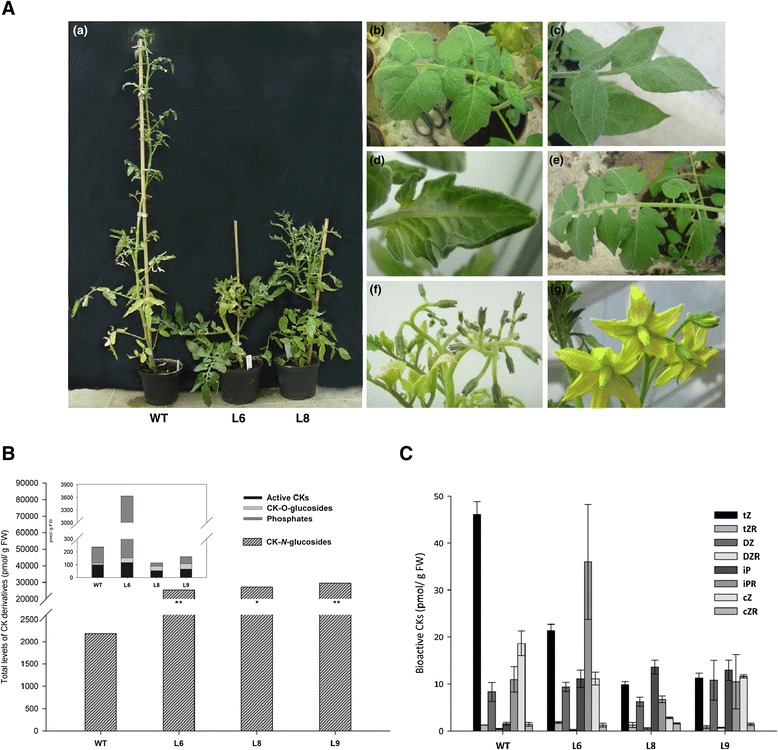
**Phenotype of T0-generation** ***35S::SlIPT3*** **tomato transformants.** Transformants showed dwarfed aerial part with a branching phenotype (a), modified leaf shape (b, c, d) and flower inflorescence (f) compared to WT plants (e and g) **(A)**. Endogenous CK levels in tomatoes divided according to the chemical structure **(B)** and contents of bioactive CKs **(C)**. WT: wild type; L6, L8 and L9: independently regenerated *35S::SlIPT3* transformants. Data represent means and SD of two replicates. *statistically significant difference datasets from WT (unpaired two-tailed Student’s *t*-test, p ≤ 0.05). **statistically significant difference from WT after Šidák correction for testing of multiple lines (multiple unpaired two-tailed Student’s *t*-test, overall α = 0.05, individual p ≤ 0.017).

The phenotype of *35S*::*SlIPT3* tomatoes substantially differed from that of the WT plants. *SlIPT3* overexpression induced plant dwarfism, release from apical dominance with a branchy phenotype, thick and ligneous stems, thicker leaves with punctual shape modification and shorter internodes (Figure 8A (a-d)). A significant yellowing of leaves, as well as accumulation of anthocyanin, was also observed. Additionally, despite inflorescence formation, no flowers were visible in most of *SlIPT3* transgenic lines (Figure 8A (f)) compared to WT plants (Figure 8A (g)). Only a single transgenic line (L1) showing the weakest phenotype (Additional file 4) was able to develop normal fruits containing seeds. Generally, during several months of cultivation, fruit formation was either significantly reduced or completely aborted. When fruits did develop, ripening was incomplete in transgenic plants. Taken together, these observations indicated that constitutive overexpression of *SlIPT3* strongly affects tomato plant development, morphogenesis and reproduction.

The strong effects of *SlIPT3* overexpression on tomato phenotype were simultaneously reflected in CK metabolism. The total CK contents extracted from young and fully expanded tomato leaves increased up to 12-fold in *35S::SlIPT3* lines (Figure 8B, Additional file 5) compared to WT plants. Proportionally, CK-*N*-glucosides represented the main group of CKs, especially in *35S::SlIPT3* lines, with iP7-glucoside (iP7G) being the predominant metabolite (Figure 8B, Additional file 5). In addition, the highest concentration of *t*Z was observed in WT plants (Figure 8C, Additional file 5). Interestingly, the CK storage forms (CK-*O*-glucosides) only slightly increased (up to 2.6-fold) in all transformants (Figure 8B). These results show that *SlIPT3* overexpression leads to a dramatic increase in CKs, particularly to the enhanced formation of iP7G throughout *N7*-glucosylation pathway, which seems to be the major metabolic pathway for bioactive CKs inactivation. Indeed, no significant differences in CK oxidase/dehydrogenase (CKX) activity were found between the control and transgenic tomatoes (Additional file 6).

### *SlIPT3* overexpression modifies cytokinin-related genes expression and plant tolerance to salinity

To elucidate the role of CK biosynthesis (*IPT*), signaling (*ARRs*) and catabolism (*CKX*) related genes in the response to *SlIPT3* overexpression in tomatoes, the expression patterns of corresponding genes were determined by qRT-PCR in young leaves of transgenic tomatoes and WT plants. In *35S::SlIPT3* lines, the transcript abundance of *SlIPT6* (which encodes a tRNA dimethylallyltransferase) was higher than in WT, whereas lower expression of *SlIPT1* was observed in all transgenic lines (Figure 7A). Likewise, upregulation of *SlARR4*, functioning in feedback regulatory loops of CKs (Figure 7B), and downregulation of CK deactivating genes *SlCKX4* and *SlCKX5* (Figure 7C) were observed in all transformants, relative to WT plants. Thus, CK-related genes expression analysis revealed upregulation of some genes involved in CK biosynthesis (*SlIPT6*) and signaling (*SlARR4*), but downregulation of genes involved in CK degradation pathways (*SlCKX4* and *SlCKX5*).

**Figure 9:**
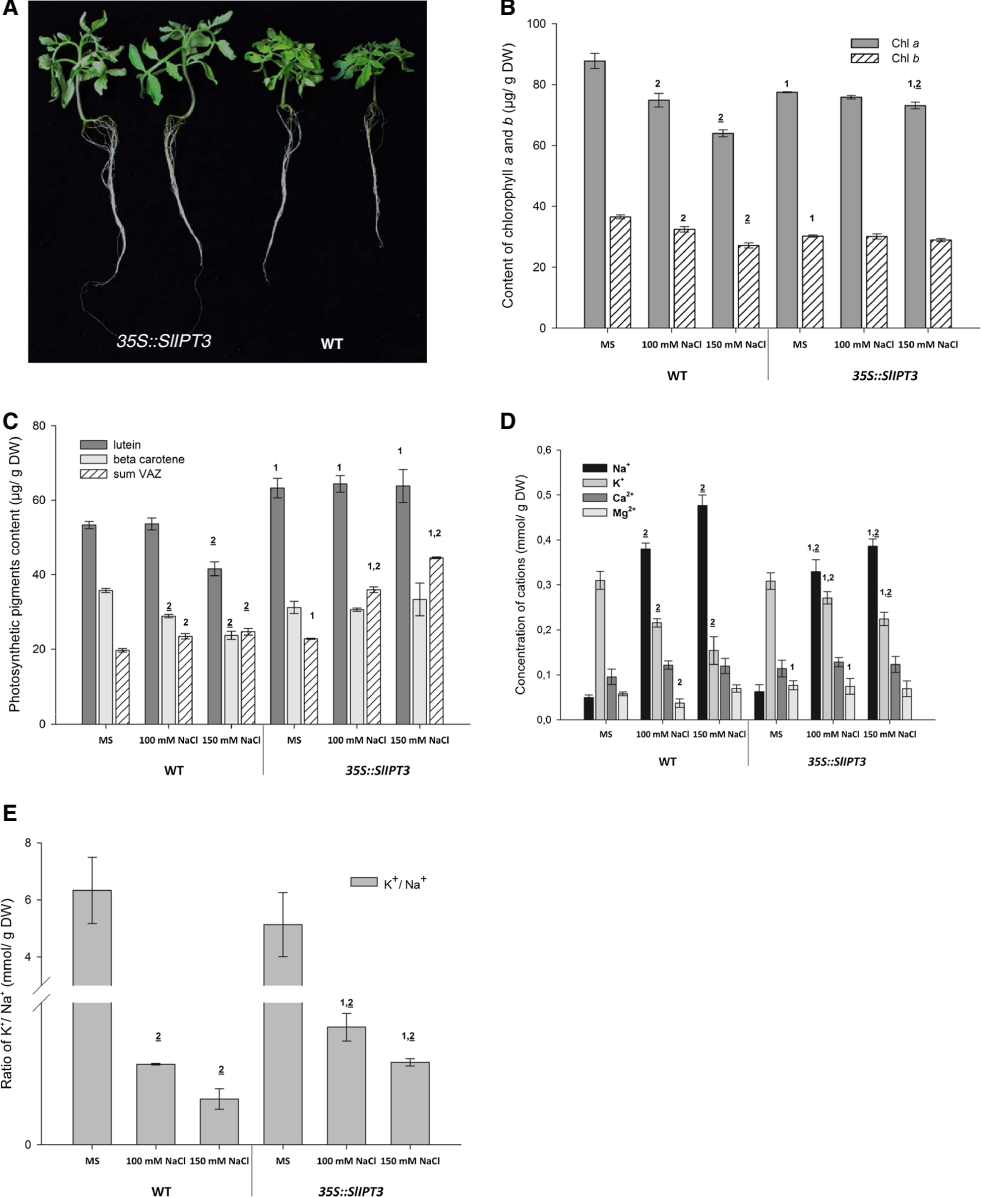
**Effect of salt stress on WT and** ***35S::SlIPT3*** **(L1) tomato plants cultivated on control and salt-containing growth media. (A)** Phenotype of WT and *35S::SlIPT3* (L1) transgenic plants grown 4 weeks on the 100 mM NaCl medium. Chlorophyll content **(B)**, carotenoid content **(C)**, cation concentration **(D)** and K^+^/Na^+^ ratio **(E)** in 4 weeks-old tomato developing leaves of WT and trangenic plants. Sum VAZ, sum violaxanthin, antheraxanthin and zeaxanthin. Data represent means and SD of two replicates for photosynthetic pigments while three replicates represent data from cation concentration. ^1^statistically significant difference from the same treatment of WT (unpaired two-tailed Student’s *t*-test, p ≤ 0.05). ^2^statistically significant difference from MS medium within the same line (unpaired two-tailed Student’s *t*-test, p ≤ 0.05), ^2^statistically significant difference from MS medium within the same line after Šidák correction for testing of multiple NaCl concentrations (multiple unpaired two-tailed Student’s *t*-test, overall α = 0.05, individual p ≤ 0.0253).

We hypothesized that the upregulation of some CK metabolic genes and enhancement of CKs content could be elementary prerequisites of tomato salt stress tolerance. As only L1 could successfully reproduce, T2 homozygous transformants from this transgenic line were further analyzed for their tolerance to salinity. For this purpose, L1 and WT lines were cultivated *in vitro* for 4 weeks on the 100 mM NaCl medium. Compared to WT plants, *35S::SlIPT3* L1 plants exhibited a larger shoot with extending leaves, whereas WT plants showed more compact shoot and smaller leaves as a result of limited growth in presence of salt (Figure 9A). Subsequently, the effects of salt stress induced either by 100 mM and 150 mM NaCl concentration on photosynthesis and nutrients balance were evaluated in WT and L1 by measurements of photosynthetic pigments and macronutrients contents.

In WT plants, chlorophyll *a* (Chl *a*) and chlorophyll *b* (Chl *b*) significantly decreased with the addition of NaCl into the growth media. In L1 plants, the content of both types of chlorophyll was significantly lower on control medium relative to WT, but remained nearly constant on NaCl containing media, and a significant decrease in Chl *a* content was only apparent on 150 mM NaCl (Figure 9B). Likewise, the levels of carotenoids (Car), especially lutein and β-carotene displaying photoprotective roles in photosynthesis, exhibited almost identical contents in *35S::SlIPT3* L1 plants regardless of growth media. In contrast, WT plants showed a significant decrease of β-carotene on both types of NaCl media, and of lutein on 150 mM NaCl. Increasing contents of Car involved in the xanthophyll cycle, violaxanthin (V), antheraxanthin (A) and zeaxanthin (Z) were as well observed in presence of salt in both WT and transgenic tomatoes, significant for WT at 150 mM NaCl and *35S::SlIPT3* grown on both concentrations of salt (Figure 9C and Additional file 7).

Shoot sodium (Na^+^), potassium (K^+^), magnesium (Mg^2+^) and calcium (Ca^2+^) concentrations were assessed in plants exposed to 0 (control), 100 mM and 150 mM NaCl (Figure 9D and E). As observed in Figure 9D, Na^+^ and K^+^ concentrations were similar in WT and L1 under control conditions of growth. Salinity induced Na^+^ accumulation and substantially reduced K^+^ contents in both types of tomato plants. However, Na^+^ accumulated to a lesser extent in L1 (0.33 and 0.37 mmol/g DW) than in WT (0.38 and 0.48 mmol/g DW) on the 100 and 150 mM NaCl medium, respectively. Similarly, K^+^ concentration was significantly higher in L1 (0.27 and 0.22 mmol/g DW) than in WT (0.216 and 0.154 mmol/g DW) in presence of 100 and 150 mM NaCl, respectively, and these results are also visible by the K^+^/Na^+^ ratio, which remained significantly higher in L1 than in WT in salt stress conditions (Figure 9E). Finally, no difference of Ca^2+^contents between WT and transgenic plants was noticed, while the Mg^2+^ concentration was significantly higher in transgenic tomatoes on control and 100 mM NaCl conditions, compared to WT (Figure 9D).

Overall, plant morphological changes in presence of salt, roughly constant levels of photosynthetic pigments, especially Chl *a*, Chl *b*, lutein and β-carotene in *35S::SlIPT3* tomatoes regardless on the growth medium and higher K^+^ contents in presence of NaCl indicate that *SlIPT3* overexpression could raise plant tolerance to salt stress.

### Both *SlIPT3* and *SlIPT4* are involved in salt stress response

**Figure 10:**
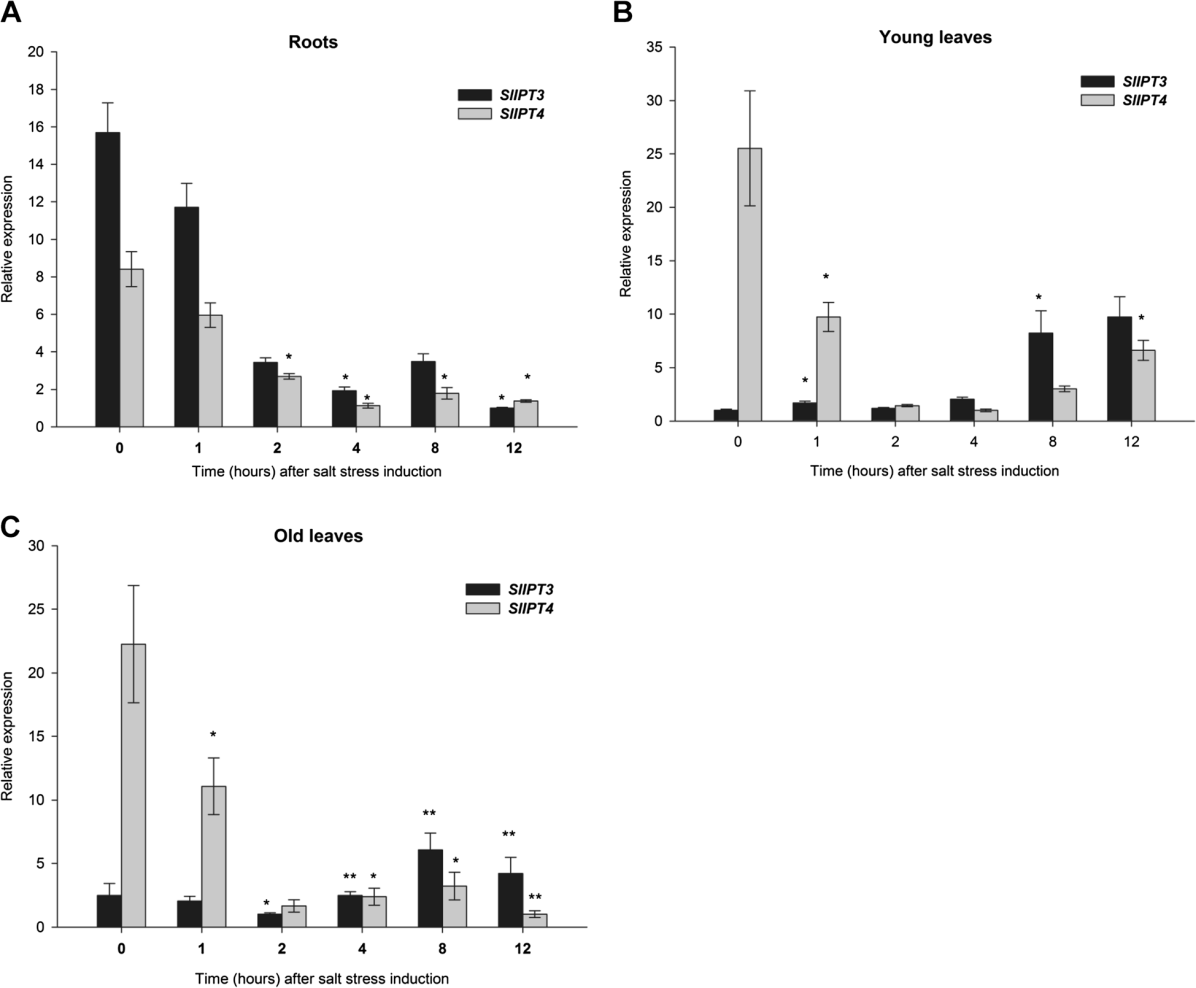
**Salinity response of** ***SlIPT3*** **and** ***SlIPT4*** **in tomato WT plants.** Analysis of *SlIPT3* and *SlIPT4* expression levels in tomato WT roots **(A)**, young leaves **(B)** and old leaves **(C)** in response to salt stress (150 mM NaCl). *Actin* and *GAPDH* were used as internal controls for normalization of *SlIPT3* and *SlIPT4* transcript levels. Data represent means and SD of two replicates. *statistically significant difference from time 0 h (unpaired two-tailed Student’s *t*-test, p ≤ 0.05). **statistically significant difference from time 0 h after Šidák correction for testing of multiple time points (multiple unpaired two-tailed Student’s *t*-test, overall α = 0.05, individual p ≤ 0.01).

To further understand the participation of *SlIPT3* and *SlIPT4* in salt stress response, their expression pattern was examined by qRT-PCR in roots, young leaves and old leaves of tomato WT plants exposed to salinity (150 mM NaCl) for 12 h. In roots (Figure 10A), which are the first organ perceiving salinity, *SlIPT3* and *SlIPT4* shared very similar expression profiles. A rapid decrease in transcript levels of both genes within 2 h after salt exposure was followed by a very small peak at 8 h for both genes, before a new decline. In young leaves, *SlIPT3* and *SlIPT4* showed partially divergent transcript accumulation profiles (Figure 10B). Indeed, *SlIPT3* transcripts gradually and regularly increased with incubation time. In contrast, *SlIPT4* was repressed 2.5-fold as rapidly as 1 h after stress initiation. This repression seemed transient, however, as a slight increase in *SlIPT4* transcripts was visible 8 h after stress onset (Figure 10B). Similar expression profiles were observed in old leaves (Figure 10C). These results indicate that *SlIPT3* and *SlIPT4* directly participate in salt stress response in tomato *via* a differential modulation of their expression, depending on the organ and duration of the stress.

## Discussion

CK homeostasis during plant development and in response to changing environmental conditions is, among other mechanisms, properly maintained by the activity of CK biosynthetic enzymes, IPTs. It was previously reported that root-localized *IPT* expression essentially improved salt stress tolerance in tomato [3]. However, the genes involved and the mechanisms of CKs regulation under salt stress conditions have not yet been elucidated. Therefore, based on functional characterization of *SlIPT3* and *SlIPT4* we provide insights into both genes involvement in the response to salt stress in tomato plants.

### Tomato SlIPT3 and SlIPT4 are non-redundant functional isopentenyltransferases

In the present study, we have demonstrated that tomato *SlIPT3* and *SlIPT4* encode two functional isopentenyltransferases (Figure 2A). Multiple alignment of proteins sequences and phylogenetic analysis revealed that SlIPT3 and SlIPT4 are closely related to Arabidopsis ortholog AtIPT3 and AtIPT5 (Figure 1B), as previously reported [12]. Interestingly, we observed identical chloroplast localization of GFP-tagged SlIPT4 protein in tomato leaf cells (Figure 3), as described in Arabidopsis where AtIPT3 was localized to plastids in roots and leaf cells [29]. These data indicate that SlIPT4 and AtIPT3 partly share similar function and protein subcellular localization. We therefore speculate that SlIPT4, and probably also SlIPT3, due to the presence of a chloroplastic transit peptide as well, may have other comparable regulatory mechanisms for protein chloroplast targeting such as farnesylation for instance, as was demonstrated for AtIPT3 [30].

Distinct spatio-temporal expression profiles of *SlIPT3* and *SlIPT4* have been observed in tomato vegetative organs and during fruit onset, development and ripening, indicating that these two genes have non-redundant functions (Figure 6A and B). In general, *SlIPT4* expression was lower relative to that of *SlIPT3* and showed its highest levels in young leaves and red fruits whereas *SlIPT3* substantially predominated in the remaining tested organs. Our data are in agreement with a previous report describing very low transcript abundance of *SlIPT4* in all tomato tested organs with flowers exhibiting the highest *SlIPT4* expression, and a greater accumulation of *SlIPT3* transcripts, especially in the very early stages of flower and fruit development [12]. Interestingly, although *SlIPT4* is less expressed than *SlIPT3* in tomato, SlIPT4 showed ten times greater activity *in vitro* than SlIPT3, which may explain why no transgenic plants could be regenerated from calli. Indeed, SlIPT4 is probably at the origin of a greater hormonal imbalance than SlIPT3, which may have impaired proper shoot and root systems regeneration.

Differences between *SlIPT3* and *SlIPT4* in organ expression specificity point out their distinct physiological roles in tomato developmental processes. However, it seems rather difficult to assign a specific role to each IPT during tomato plant development, due to their overlapping functions. Indeed, although *AtIPT3* is one of the three most expressed *IPT* genes in *Arabidopsis* during the vegetative phase, *ipt3* knock-out mutant exhibited no distinguishable phenotype from WT plants, indicating functional redundancy between the Arabidopsis IPT proteins [17]. Therefore, none of the visible phenotypes or characteristics in *SlIPT3* and *SlIPT4* complemented Arabidopsis *ipt3* knock-out mutants were apparent in comparison to WT plants under control conditions (Additional file 1, Figure 4A and B).

### Ectopic expression of *SlIPT3* alters tomato phenotype and CK metabolism

Overexpression of *SlIPT3* under *35S CaMV* promoter and consequent CK over-accumulation had extensive negative effects on tomato growth under control conditions (Figure 8), resulting in dwarf phenotype and delayed senescence, substantially limiting formation of tomato generative organs. Only one transgenic line (L1) showing the weakest phenotypic differences from WT was able to produce seeds (Additional file 4). A similar effect of CK overabundance in flowering delay was reported in stably transformed tobacco plants with *35S*::*MdIPT3a* [31]. Likewise, phenotype alterations such as a strong delay in growth, shorter rosettes, serrated leaves and decreased apical dominance were observed in Arabidopsis plants overexpressing maize *ZmIPT2* [32]. In contrast, no changes in flowering time were evident in Arabidopsis and tobacco transgenic plants expressing *IPT* under *HSP70* [33], underlining the crucial role of CKs accumulation and homeostasis during flowering.

We found out that constitutive expression of *SlIPT3* increased CK production in tomatoes by up to 12-fold relative to WT plants, primarily iP7G (Figure 8B, Additional file 5). Similarly, high concentrations of iP7G in Arabidopsis complemented plants cultivated on both control and salt media suggest that the formation of CK-*N7*-glucosides may be a sufficient pathway for downregulation of bioactive CKs because no significant enhancement of CKX activity was detected in *35S*::*SlIPT3* tomatoes when compared to WT (Additional file 6). In accordance with our results, *IPT* overexpression resulted in increased levels of bioactive iP, *t*Z and their ribosides in tomato [3] and tobacco [31,32]. However, detailed data regarding all CK derivatives were not reported in these previous studies. Based on the observation that iPRMP/iPRDP/iPRTP are the first products in CK biosynthesis [9,10], that iP and iPR are the best substrates for CKX activity [34] and are in massive amounts metabolized to CK-*N*-glucoside forms (Additional file 5), we conclude that iP-type CKs have a prominent role in fast regulation and balancing of the CK metabolism.

### SlIPT3 and SlIPT4 participate in salinity response in tomato and Arabidopsis

Expression analyses of *SlIPT3* and *SlIPT4* in tomato vegetative organs confirmed the participation of both genes in response to salt stress. After stress onset, salinity evoked repression of *SlIPT3* and *SlIPT4* in roots, and differently affected *SlIPT3* and *SlIPT4* in young and old leaves. After 6 to 8 h of exposure to salinity, increasing levels of both transcripts were detected in young leaves, suggesting preferential CK biosynthesis in photosynthetic organs and thus underlining CKs stress-prevention and anti-senescence properties (Figure 10B). Similarly, an augmentation of *IPT* expression was apparent both in maize leaves and roots following longer exposure to salinity whereas shorter salt treatment (30 min) induced a repression of *IPT* encoding genes (3 out of 4) in maize leaves [11]. Likewise, two weeks-old Arabidopsis plants showed downregulation of *AtIPT1* and *AtIPT3* as early as 1 h after salt treatment, whereas the expression of *AtIPT5* and *AtIPT7* was elevated. Conversely, with longer salt stress, the majority of *AtIPT* transcripts were repressed [35]. Together, these data indicate a dynamic regulation of CK biosynthesis/metabolism over the stress period.

Moreover, multiple putative stress-responsive *cis*-regulatory elements were identified in the promoter regions of *BrIPTs* (and *BrCKXs*) promoters, pointing to the response of these genes to stress stimuli and a possible response to unfavourable conditions *via* changing CK status [36]. Similarly in this study, an *in silico* analysis of *SlIPT3* and *SlIPT4* promoters [37] (www.dna.affrc.go.jp/PLACE/index.html) revealed the presence of putative stress response elements and many ARR binding *cis-*elements, indicating at least for *SlIPT3* the existence of salt stress tolerance mechanisms similar to those in *BrIPTs*. Our findings in tomato plants illustrated that the regulation of *SlIPT3* and *SlIPT4* in response to salt stress is organ specific, depends on the duration of stress exposure and potentially also on the presence of *cis*-regulatory elements in the promoters of CK-related genes. In addition, diverse roles of both tomato *SlIPT*s in salt stress responses have been characterized in Arabidopsis *ipt3* KO complemented plants under longer period of salt treatment (Figures 4 and 5).

Transgenic plants ectopically expressing *SlIPT3* under *35S* promoter showed superior above-ground growth after several weeks on salt medium, relative to WT plants (Figure 9A). Beneficial effects of CKs overproduction in response to salt stress were reported in tomato [3]. CKs elevation enhanced shoot growth and prevented senescence under salinity conditions due to the specific induction of *IPT* in roots by a *heat-shock* promoter (*HSP70*::*IPT*). Likewise, higher CK concentration was associated with salt tolerance in tomatoes overexpressing a cysteine-2/histidine-2-type zinc finger transcription factor [38]. Similarly, expression of *Agrobacterium tumefaciens IPT* under the stress inducible promoter *rd29A* resulted in higher salinity tolerance in transgenic tobacco [23]. Using specific promoters for *IPT* overexpression also successfully improved tolerance to drought in rice [39], peanut [40], cassava [41], tobacco [42] and to the cold stress in tobacco [43]. On the other hand, tobacco plants with reduced CK contents showed smaller osmotic potential and higher drought resistance than WT plants [44]. Likewise, Arabidopsis knock-out mutants for two CK histidine kinase receptors (*ahk2* and *ahk3*) showed tolerance to drought and salinity [45]. Aspects of plant phenotype, such as root architecture modifications and growth inhibition in transgenic plants, influenced by imbalance between CKs accumulation and distribution can partly explain tolerance to various environmental stresses. Thus, root-specific *CKX* overexpression enhanced root system development and consequently increased drought tolerance, nutrient uptake and leaf nutrient enrichment in *Arabidopsis* [43,46]. Consequently, it is still difficult to estimate the precise nature of CK effects due to the complexity of phytohormones metabolism and other cross-talk networks.

Salinity tolerance of *35S::SlIPT3* plants is correlated, among others, with improved nutrients balance and retention of the photosynthesis capacity, in addition to the senescence-protective effects of the photosynthetic pigments (Figure 9). Indeed, salinity strongly impacts tomato plants ionic status, as Na^+^ concentration considerably increases in leaves, inducing a premature senescence, while K^+^ content differently declines depending on the leaf age [4]. Transgenic tomato plants *HSP70::IPT* showing improved growth and final yield under long-term salinity treatment consequently accumulate less Na^+^ in leaves and roots than WT, but higher K^+^ contents, underlining the importance of the limitation of toxic ions accumulation for plant development [3]. In addition, salinity strongly affects plant photosynthesis, and salt-induced reduction in photosynthetic pigments such as Chl and Car have been reported in several studies [47]. Variations in Chl and Car contents under different salt concentrations and time exposure were observed in three varieties of tomato cultivars, suggesting that sensitivity to salt stress depends on plant genotype [48]. Our results indicate that the contents of Chl *a* and Ch *b* as well as lutein and β-carotene are maintained in *35S::SlIPT3* plants at the same levels regardless of the growth media in contrast to WT, in which salt treatments decreased both Chl and Car contents (Figure 9B and C). Similarly, transgenic tomatoes overexpressing *IPT* within the root zone maintained their photosynthesis ability under long-term salinity treatement [3], while transgenic tobacco expressing stress-inducible promoter *rd29A-IPT* showed only slight decrease in Chl contents in comparison to WT plants [23]. Significant accumulation of pigments involved in the xanthophyll cycle in *35S::SlIPT3* plants exposed to salt may reflect higher intensity in photoprotective function of Car in transgenic plants than in WT plants.

In summary, our results indicate that constitutive expression of *SlIPT3* induces several changes in plant growth, nutrients accumulation, hormonal metabolism and photosynthesis maintenance, leading to enhanced tolerance to salt stress.

### Mechanism of SlIPT3 and SlIPT4 regulation under salt and CK treatments

The comprehensive mechanism of CK regulatory feedback loop is supported by our findings that exogenous application of *t*Z inhibited the expression of *SlIPT3* and *SlIPT4* within 4 h, enhanced the expression of negative-feedback regulator type-A *SlARR4* and slightly moderated the expression levels of CK primary response genes type-B *SlARR1* and *SlARR12* (Figure 6C and D). Comparable data reporting downregulation of the *IPT* genes after CK treatment have been reported for Chinese cabbage [36] and maize [11]. The connection between CK homeostasis maintenance *via* type-B response regulators and salinity response was reported in Arabidopsis where ARR1 and ARR2 were shown to control sodium accumulation in shoots by regulating expression of genes encoding high-affinity K^+^ transporter 1;1 responsible for Na^+^ exclusion from the root xylem [25]. These data suggest that CK homeostasis may be effectively regulated, not only by CKs molecules, but also environmental stimuli.

Taken together, we have shown the mechanism of *SlIPT3* and *SlIPT4* response to early salt stress and their feedback regulation by exogenous CK treatment in tomato plants. Based on the data obtained in this study, we propose a model illustrating the complexity of CK networks in tomato plants in response to salinity (Additional file 8). In this model, we only included relations supported by our experimental data. We outline an organ specific down-regulation of *SlIPT3* and *SlIPT4* transcripts immediately after stress onset, followed by transcripts increase with the duration of the stress treatment. The complexity of CK networks is demonstrated by the repression of *SlIPT3* and *SlIPT4* after exogenous application of *t*Z, while an overabundance of CKs (especially iP7G) in *35S*::*SlIPT3* may ensure a fitter phenotype under salt stress conditions. Similarly, the response to salinity by *SlIPT3* and *SlIPT4* was paralleled in Arabidopsis *ipt3* KO complemented plants where salinity resulted in elevation of CKs (mainly *N*-glucosides) with successive downregulation of *AtIPTs* to maintain CK homeostasis (Figure 5, Additional file 8).

## Conclusions

In summary, we have characterized *SlIPT3* and *SlIPT4* response to early salt stress in tomato, and identified *SlIPT3* as a key player in the CK metabolism of tomato plants under salinity conditions. Our results contribute to the understanding of CK regulation at the molecular level and provide a potentially useful tool to obtain and improve high-quality stress-tolerant crops in agriculture.

## Methods

### Plant materials and growth conditions

Tomato (*Solanum lycopersicum* L. cv Ailsa Craig) seeds were pre-germinated on Whatman 3MM paper soaked with sterile water in a sterile petri dish until the radicle was a few mm long. Seeds were sown in trays filled with a perlite-vermiculite mix (1/3, v/v), as previously described [4]. Four weeks later, tomato plantlets cultivated in a growth chamber with a 16 h light (24°C)/8 h dark (22°C) photoperiod were transferred into the 52 L tanks containing aereted half-strength modified Hoagland nutrient solution, and kept in hydroponic system for an additional ten days before applying the salt stress, as previously described [38].

Salt stress treatment of wild-type tomatoes was evoked by direct addition of 150 mM NaCl into the culture tank. For hormonal assays, plants were grown in smaller tanks with a capacity of 4.5 L. Zeatin (mixed *cis*- and *trans*-isomers including approximately 80% of *trans*-zeatin, Sigma Z0164) was used at a final concentration of 10 μM. In order to investigate the spatio-temporal expression profiles of *SlIPT3* and *SlIPT4*, plants were grown in soil in the greenhouse during 8 weeks. Three plants were collected for each time point of the experiment (0 h, 1 h, 2 h, 4 h, 8 h and 12 h). After harvesting, samples were immediately frozen in liquid nitrogen and stored at −80°C until further analyses. For tomato *35S::SlIPT3* (L1) *in vitro* culture, seeds were surface sterilized with a mixture of 2.5% (v/v) potassium hypochlorite and 0.02% (v/v) Triton X-100 for 15 min and rinsed 5 times with sterile water. Seeds were sown on full MS media and the medium for transgenic line selection was supplemented with kanamycin (100 mg/L). Two weeks later, plants were transferred into magenta boxes on the control medium and the medium containing NaCl (100 mM) and cultivated under the same conditions as before (16 h light (24°C)/8 h dark (22°C) photoperiod in the growth chamber). Phenotypic characteristics were evaluated after four weeks.

*Arabidopsis thaliana* ecotype Columbia was used as the wild-type for all Arabidopsis experiments. The *ipt3* T-DNA insertion line N553810 was obtained from the Nottingham Arabidopsis Stock Center. Seeds were surface sterilized as described for tomato and kept at 4°C for 48 h before being sown on Petri dishes containing half-strength MS with 0.9% (w/v) agar. Additionally, the medium for *ipt3* KO mutants selection contained 35 mg/L kanamycin and *SlIPT3* and *SlIPT4* Arabidopsis complemented plants were selected on the medium supplemented with 15 mg/L hygromycin. Plates were then transferred to the growth chamber with a 16 h light (20°C)/8 h dark (18°C) photoperiod. For the analysis of salt stress tolerance, half-strength MS medium containing 100 mM NaCl was used. The root length (from hypocotyl base to the root tip) of at least twenty vertically growing ten-days-old plants was measured. Differences in root length were tested for significance using a Mann–Whitney U test and data representing two biological replicates. Germination percentage was determined for eighteen seedlings after 24 h using a magnifying glass, when the radicle occurs. Survival percentage of eighteen seedlings was evaluated in thirteen-day-old plants. Total numbers of germinated and non-germinated seeds (54) were cross tabulated for each pair of groups and Fisher’s exact test was then applied to the resulting 2x2 contingency tables. For both assays, the data represent three biological replicates.

### Plasmid constructions and plant transformation

*SlIPT3*, *SlIPT4, SlARR1* (XP_004239797)*, SlARR4* (XP_004238726) and *SlARR12* (XP_004251765) cDNAs were amplified with the *Pfu* DNA polymerase (Promega) in a final reaction volume of 50 μL following the manufacturer’s instructions, using 300 ng of cDNAs synthesized from total RNA extracted from tomato grown *in vitro* as template. Specific primers used to amplify the coding sequence of *SlIPT3* and *SlIPT4* contain anchors: forward (5′-GGGGACAAGTTTGTACAAAAAAGCAGGCT-3′) and reverse (5′-GGGGACCACTTTGTACAAGAAAGCTGGGT-3′) allowing BP cloning (Invitrogen) in the pDONR221 vector. Specific primers for every coding sequence were as follows: IPT3F (5′-TATGAATATTGTGTTACAACATATTG-3′), IPT3R (5′-CTAGTGCGTCATAGTAGCAAC-3′), IPT4F (5′-ATGATTGGCATGATGAACTCT-3′), IPT4R (5′-TTAATAGTGAGATGCTGCTGCC-3′). The PCR product was then transferred to the pDONR221 entry vector (Invitrogen) by a BP recombination reaction prior to DNA sequencing (Macrogen). *SlIPT3* and *SlIPT4* were subsequently transferred either to the pK7WG2D or to the pH7WG2D binary vectors [49]. *SlARR* coding sequences were amplified with the following primers: ARR1F (5′-AAAAAATTCAAAATTTTGAAGAAAGT-3′) and ARR1R (5′-AACTACGCTCTTTTCGCATC-3′), ARR4F (5′-ATTTGGTGAAATATTTGTGGGTT-3′) and ARR4R (5′-GCTTAAACGACCCCGGAAGTA-3′), ARR12F (5′-ATGACTGTGGAGGAAATTAGA-3′) and ARR12R (5′-TCATAAACCTGAACCAAGTGAA-3′), prior to cloning into the pGEM-T-easy vector (Promega).

*SlIPT3* and *SlIPT4* constructs were introduced into the *Agrobacterium tumefaciens* strain GV3101 for Arabidopsis transformation and in the LBA44A4 for tomato transformation. Competent cells were prepared as previously described [50]. For subcellular localization, *SlIPT3* and *SlIPT4* cDNA sequences lacking a stop codon were cloned in frame with the *GFP* sequence in the pK7FWG2 binary vector [49].

Tomato stable transformation was adapted from a published method [51] and tomato genomic DNA extraction was performed as described before [52].

Arabidopsis plants were transformed by the floral dip method [53]. For Arabidopsis *ipt3* complementation, a combination of 3 primers LBb1.3 (5′-ATTTTGCCGATTTCGGAAC-3′), IPT3LP (5′-TGGAATGGTTGAGGAAGTCAG-3′), IPT3RP (5′-CATTGGCTTAGAAATTTGTGTCC-3′) was used to identify homozygous plants in the segregating F3 population.

### RNA extraction and real-time PCR analysis

Tomato total RNAs were extracted from all tissues, treated with the RNase-free DNase I (Promega) and purified according to a previous reference [54]. An aliquot of 2 μg was subsequently used as a template for reverse-transcription (RevertAid™ H Minus First Strand cDNA Synthesis Kit, Fermentas) using oligo d(T)_18_ according to the instructions in the manual. Transcript levels of the different genes were measured by qRT-PCR using SYBR Green on a LightCycler 480 II (Roche). PCR reactions were performed in triplicate using 0.2 μM of each primer, 5 μL SYBR Green mix (Promega), and 300 ng of DNAse-treated cDNA in a final volume of 10 μL. Negative controls were included in each run. PCR conditions were: initial denaturation at 95°C for 120 s followed by 45 cycles of 95°C for 10 s, and 58°C for 15 s. Amplification was followed by melting curve analysis to check the specificity of each reaction. The primers IPT3PCRQF (5′-CCTTCTTGCACAAAGTTGCT-3′) and IPT3PCRQR (5′-TGAGGTTATTGATATTAGCAAATA-3′) were used to amplify a 107 bp sequence, while IPT4PCRQF (5′-GGACAGAGCAGAAAGAT-3′) and IPT4PCRQR (5′-TAATAGTGAGATGCTGCTGCCA-3′) allowed amplification a 108 bp PCR product.

Data obtained from tomato were normalized according to *SlGAPDH* and *SlActin* expression levels. The primers used were GAPDHF (5′-GGTGCCAAGAAGGTTGTGAT-3′) and GAPDHR (5′-TTTTCTGGGTGGCAGTCAT-3′) that generated a 217 bp PCR product, ActinF (5′-ATGGTGGGTATGGGTCAAAA-3′) and ActinR (5′-GAGGACAGGATGCTCCTCAG-3′) that allowed the formation of a 183 bp PCR product. Normalized expression of *SlIPT3* and *SlIPT4* was calculated using the Gene Expression Analysis for iCycle iQ_ Real Time PCR Detection System software from Bio-Rad with a method derived from the algorithms previously outlined [55].

A similar procedure was adopted for the qRT-PCR analysis of Arabidopsis samples. The list of primers used for both tomato and Arabidopsis qRT-PCR analysis is attached as Supplementary data (Additional files 9 and 10). Statistical differences in target gene transcripts were evaluated using unpaired Student’s *t*-test p ≤ 0.05 to compare ΔCt values.

### *In vitro* isopentenyltransferase activity assay

*SlIPT3* and *SlIPT4* coding sequence were respectively amplified by PCR with the following primers: SlIPT3F (5′- GGATCCTAATACGACTCACTATAGGGAACAGCCACCATGAATATTGTGTTACAACATATT-3′) and SlIPT3R (5′-TTTTTTTTTTTTTTTTTTTTTTTTTTTTTTTTAGTGCGTCATAGTAGCAA-3′) SlIPT4F (5′- GGATCCTAATACGACTCACTATAGGGAACAGCCACCATGATTGGCATGATGAACTCT −3′), SlIPT4R (5′- TTTTTTTTTTTTTTTTTTTTTTTTTTTTTTTTAATAGTGAGATGCTGCTG-3′). The forward primer included a sequence of the T7 RNA polymerase promoter, while the reverse primer contained a stop codon and a polyadenylated extremity. Generated amplicons were subcloned into pGEMT-T Easy vector (Promega) before sequencing. 800 ng of purified PCR product were used for *in vitro* protein translation in rabbit reticulocyte cells (TnT® T7 Quick Coupled Transcription/Translation System, Promega), following the manufacturer’s instructions, in a final reaction volume of 50 μL.

SlIPT3 and SlIPT4 were examined for DMAPP:[^3^H]AMP, DMAPP: [^3^H]ADP and DMAPP: [^3^H]ATP isopentenyltransferase activity using an adapted method [9,32]. The reaction mixture, consisting of 50 μL protein produced *in vitro* as described above, 100 μL of salts and buffer containing 37.50 mM KCl, 5 mM MgCl_2_ and 12.5 mM Tris–HCl (pH 7.5) and 100 μL of substrates DMAPP (34 nmol) plus [^3^H]AMP, [^3^H]ADP or [^3^H]ATP (each 1 nmol, 20 Ci/mmol) was incubated at 30°C for 1 h, 2 h, 8 h, 24 h and 48 h. Negative controls included either a crude extract of non-transformed rabbit reticulocyte cells or no cells at all. To stop the reaction and to precipitate the SlIPT3 or SlIPT4 proteins, cold ethanol (150 μL) was added into an aliquot of 50 μL of reaction mixture at the indicated times. The samples were vortexed and stored at −20°C for 2 h. The mixture was then centrifuged for 25 min, 20 000 g at 4°C. The supernatant was recovered in clean tubes and evaporated in a vacuum concentrator (Alpha RVC, Christ) to dryness. The pellet was resuspended in 50 μL of 5% MeOH. Each sample was analyzed by HPLC (Perkin Elmer) coupled to a radioactivity flow detector (Ramona 2000, Raytest). The radioactive metabolites were identified on the basis of comparison of their retention times with authentication standards. IPT activity was determined in four independent experiments, which showed the same tendencies although with different absolute values (Additional files 11). Therefore the results of one representative experiment are presented.

### Cytokinins extraction and quantification

Endogenous CKs were extracted from homogenized young fully expanded tomato leaves (15 mg of dry weight) and 17 DAS Arabidopsis plants (150 mg of fresh weight) using an extraction buffer consisting of methanol/formic acid/water (15/1/4, v/v/v) according to the published method [56]. Following the addition of stable isotope labeled internal standards (10 pmols), samples were extracted for 1 h at −20°C. The solids were separated by centrifugation (20 000 g, 20 min, 4°C) with a subsequent collection of supernatants. The pellets were re-extracted with an additional 0.5 mL of extraction buffer (30 min at −20°C). The supernatants were collected after re-centrifugation, and incubated 30 min at −80°C. Samples were evaporated in a vacuum concentrator (Alpha RVC, Christ), re-dissolved in 0.5 mL of 1 M formic acid and applied to a mixed mode reversed phase-cation exchange SPE column (Oasis-MCX, Waters).

The CK fraction was sequentially eluted with 0.35 M NH_4_OH in 60% methanol. This fraction was evaporated to dryness in a vacuum concentrator and dissolved in 5% MeOH. An aliquot (0.01 mL) from each sample was separately analyzed on a high-performance liquid chromatography (HPLC) (Ultimate 3000, Dionex) coupled to a hybrid triple quadrupole/linear ion trap mass spectrometer (3200 Q TRAP, Applied Biosystems) using a multilevel calibration graph with [^2^H]-labeled internal standards as described before [57,58]. Data are presented as mean ± standard error.

### CKX activity assay *in vitro*

CKX enzymes were extracted and partially purified from the same tomato leaves samples used for hormones extraction (WT and *35S::SlIPT3*) according to a published reference [59]. CKX activity was determined by *in vitro* assays based on the conversion of [2-^3^H]iP (prepared by the Isotope Laboratory, IEB ASCR, Prague, Czech Republic) to [^3^H]adenine and expressed as pmol adenine mg protein^−1^ h^−1^. The CKX activity was determined in two biological replicates for each variant.

### Photosynthetic pigments and ion extraction, quantification and analysis

The content of photosynthetic pigments (Chl *a* and Chl *b*, β-carotene, lutein, neoxanthin, violaxanthin, zeaxanthin and antheraxanthin) was determined in acetone extracts made from the lyophilized developing leaves of 4 weeks-old plants analyzed by a high-performance liquid chromatography (ECOM, Czech Republic). The analysis was made using a reversed phase column (Watrex Nucleosil 120 5 C18, 5 μm particle size, 125 × 4 mm, ECOM, Czech Republic). The solvent system comprised acetonitrile/methanol/water (80/12/10, v/v/v) followed by methanol/ethylacetate (95/5, v/v), the total analysis time was 25 min, and the linear gradient was run from 2 to 6 min (the flow rate was 1 cm^3^ min^−1^, the detection wavelength was 445 nm). Data were captured and calculated by PC-software Clarity (DataApex, Czech Republic). The photosynthetic pigments were determined in two biological replicates of each variant measured independently by two times.

Shoot mineral quantification was conducted on the same samples as for pigments analysis using an atomic absorption spectrometer (Thermo Scientific ICE3300) as described previously [38].

### Availability of supporting data

Phylogenetic data are available in Treebase (www.treebase.org) database under submission identity 16841.

## Additional files

Additional file 1:
**Phenotype of 5 DAS Arabidopsis WT,** ***ipt3*** **KO, and** ***SlIPT3*** **or** ***SlIPT4*** **complemented plants cultivated on control medium.**
Additional file 2:
**Endogenous CKs content (pmol/g FW) of 17 DAS Arabidopsis** ***ipt3*** **plants complemented with** ***SlIPT3*** **or** ***SlIPT4*** **and grown on control medium.** The system of abbreviations was adopted and modified according to published reference [60]. Additional file 3:
**Endogenous CKs content (pmol/g FW) of 17 DAS Arabidopsis** ***ipt3*** **plants complemented with** ***SlIPT3*** **or** ***SlIPT4*** **and grown on salt medium (100 mM NaCl).** The system of abbreviations was adopted and modified according to published reference [60]. Additional file 4:
**Phenotype of T1-generation tomatoes** ***35::SlIPT3*** **Line 1 (L1) transformants.**
Additional file 5:
**Endogenous CKs content (pmol/g FW) in young leaves of T0** ***35S::SlIPT3*** **tomatoes.** The system of abbreviations was adopted and modified according to published reference [60]. Additional file 6:
**Cytokinin oxidase/dehydrogenase (CKX) activity in** ***35S::SlIPT3*** **tomato young leaves (L6 and L7).**
Additional file 7:
**The content of photosynthetic pigments (μg/g DW) in 4 weeks-old tomato developing leaves of WT and T2-generation of** ***35S::SlIPT3*** **(L1) tomato plants cultivated on the control and salt growth media.** DEPS (De-epoxidation state) is calculated according to formula: DEPS = (0.5*A + Z)/(V + A + Z); DEPS (De-epoxidation state per chlorophyll) is calculated according to formula: DEPSC = (0.5*A + Z)/(Chl *a* + *b*). ^ expressed in relative unit. *Data represent means and SD of two replicates. *significantly different datasets from WT (unpaired Student's *t*-test, P ≤ 0.05). Additional file 8:
**Hypothetical scheme of** ***SlIPT3*** **and** ***SlIPT4*** **response to early salt stress and the feedback regulation by exogenous CK treatment in tomato plants.** After salt stress (150 mM NaCl) treatment, immediate down-regulation of *SlIPT3* and *SlIPT4* transcripts with a subsequent up-regulation in tomato vegetative organs was determined. Repression of both genes after exogenous application of *t*Z demonstrated the complexity of CK networks, while the overabundance of CKs (specially iP7G) in *35S*::*SlIPT3* may ensure a stronger phenotype under salt stress (100 mM NaCl) conditions. Additional file 9:
**Sequences of tomato primers used for qRT-PCR analysis.**
Additional file 10:
**Sequences of Arabidopsis primers used for qRT-PCR analysis.**
Additional file 11:
***In vitro*** **enzymatic activity of SlIPT3 and SlIPT4.** Conversion of non-labeled AMP added into the water and cells containing SlIPT3 (A) or cells containing SlIPT4 (B) measured by IP-HPLC with UV detector. Conversion of radioactive labeled precursors AMP, ADP and ATP added into the water and cells containing SlIPT3 or SlIPT4 were measured by LCMS (C).
