# Supplementary material for: Tomato (Solanum lycopersicum L.) SlIPT3 and SlIPT4 isopentenyltransferases mediate salt stress response in tomato
Source: BMC Plant Biol. 2015 Mar 12;15:85. doi: 10.1186/s12870-015-0415-7 (PMC4404076; doi:10.1186/s12870-015-0415-7)
Supplement: Additional file 1: — Phenotype of 5 DAS Arabidopsis WT, ipt3 KO, and SlIPT3 or SlIPT4 complemented plants cultivated on control medium. [file 12870_2015_415_MOESM1_ESM.pptx]

## Slide 1
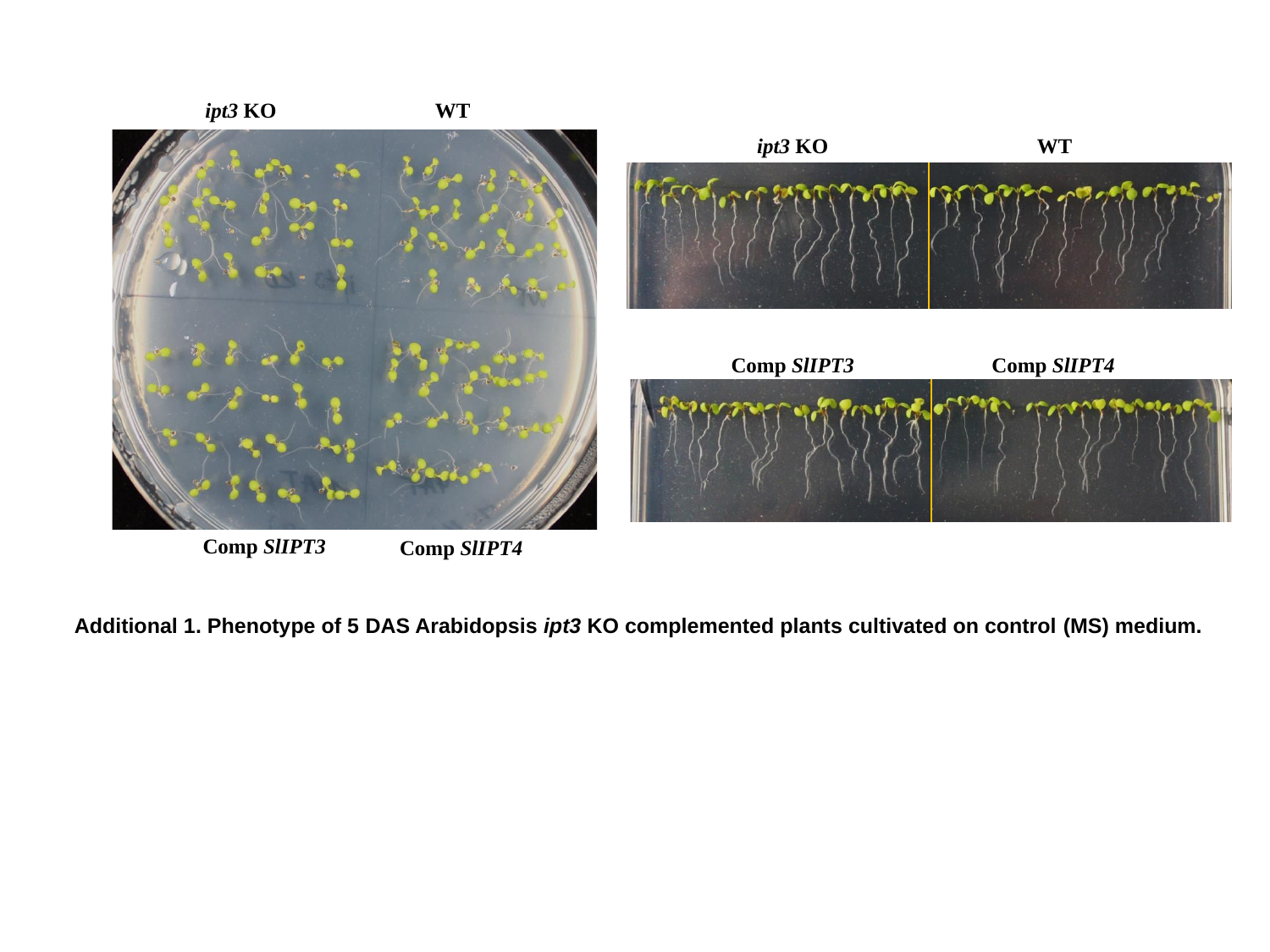

ipt3 KO
WT
Comp SlIPT3
Comp SlIPT4
ipt3 KO
WT
Comp SlIPT3
Comp SlIPT4
Additional 1. Phenotype of 5 DAS Arabidopsis ipt3 KO complemented plants cultivated on control (MS) medium.
