# Supplementary material for: Tomato (Solanum lycopersicum L.) SlIPT3 and SlIPT4 isopentenyltransferases mediate salt stress response in tomato
Source: BMC Plant Biol. 2015 Mar 12;15:85. doi: 10.1186/s12870-015-0415-7 (PMC4404076; doi:10.1186/s12870-015-0415-7)
Supplement: Additional file 2: — Endogenous CKs content (pmol/g FW) of 17 DAS Arabidopsis ipt3 plants complemented with SlIPT3 or SlIPT4 and grown on control medium. The system of abbreviations was adopted and modified according to published reference [60]. [file 12870_2015_415_MOESM2_ESM.pptx]

## Slide 1
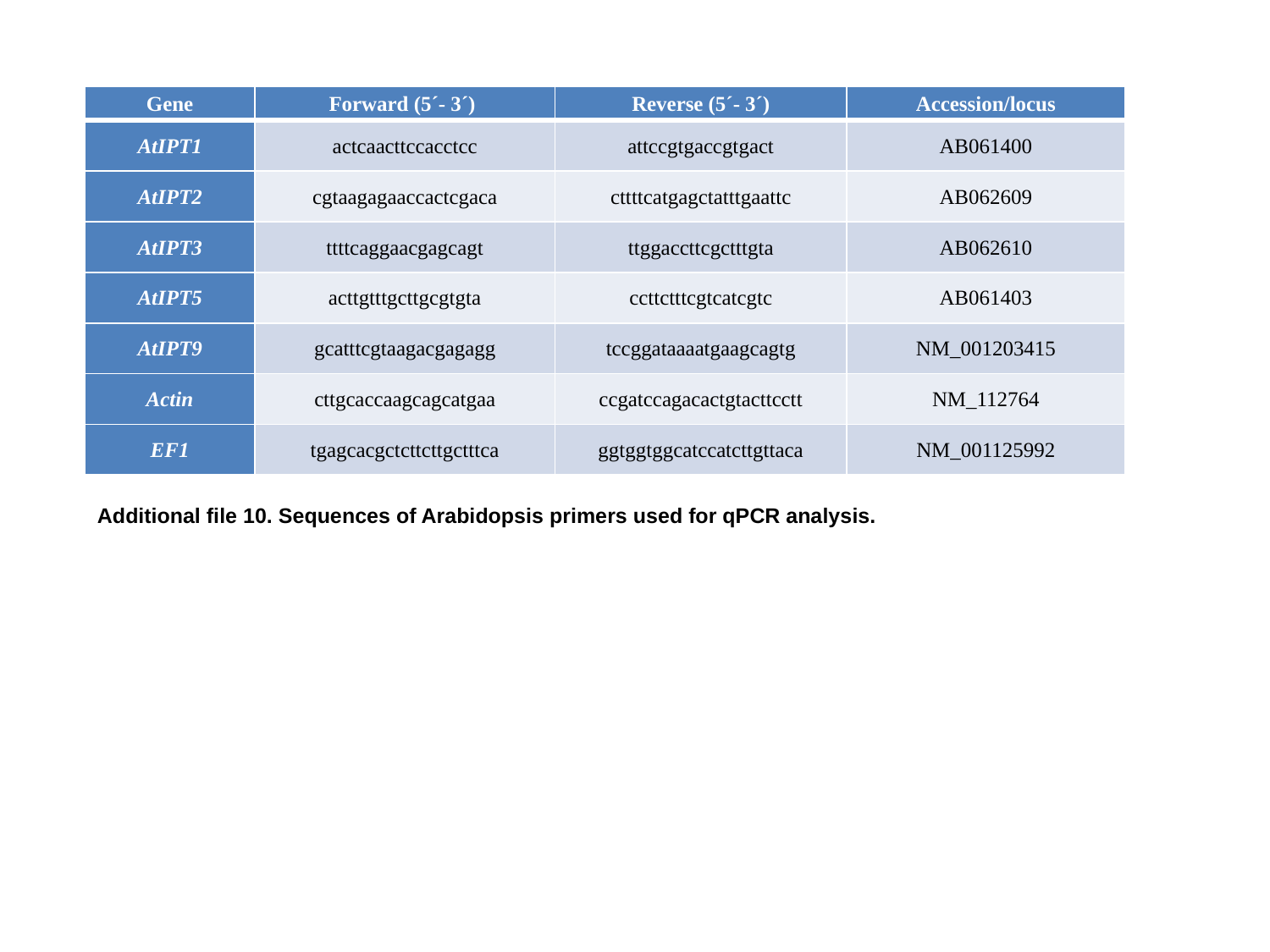

| Gene | Forward (5´- 3´) | Reverse (5´- 3´) | Accession/locus |
| --- | --- | --- | --- |
| AtIPT1 | actcaacttccacctcc | attccgtgaccgtgact | AB061400 |
| AtIPT2 | cgtaagagaaccactcgaca | cttttcatgagctatttgaattc | AB062609 |
| AtIPT3 | ttttcaggaacgagcagt | ttggaccttcgctttgta | AB062610 |
| AtIPT5 | acttgtttgcttgcgtgta | ccttctttcgtcatcgtc | AB061403 |
| AtIPT9 | gcatttcgtaagacgagagg | tccggataaaatgaagcagtg | NM\_001203415 |
| Actin | cttgcaccaagcagcatgaa | ccgatccagacactgtacttcctt | NM\_112764 |
| EF1 | tgagcacgctcttcttgctttca | ggtggtggcatccatcttgttaca | NM\_001125992 |
Additional file 10. Sequences of Arabidopsis primers used for qPCR analysis.
