# Supplementary material for: Tomato (Solanum lycopersicum L.) SlIPT3 and SlIPT4 isopentenyltransferases mediate salt stress response in tomato
Source: BMC Plant Biol. 2015 Mar 12;15:85. doi: 10.1186/s12870-015-0415-7 (PMC4404076; doi:10.1186/s12870-015-0415-7)
Supplement: Additional file 3: — Endogenous CKs content (pmol/g FW) of 17 DAS Arabidopsis ipt3 plants complemented with SlIPT3 or SlIPT4 and grown on salt medium (100 mM NaCl). The system of abbreviations was adopted and modified according to published reference [60]. [file 12870_2015_415_MOESM3_ESM.pptx]

## Slide 1
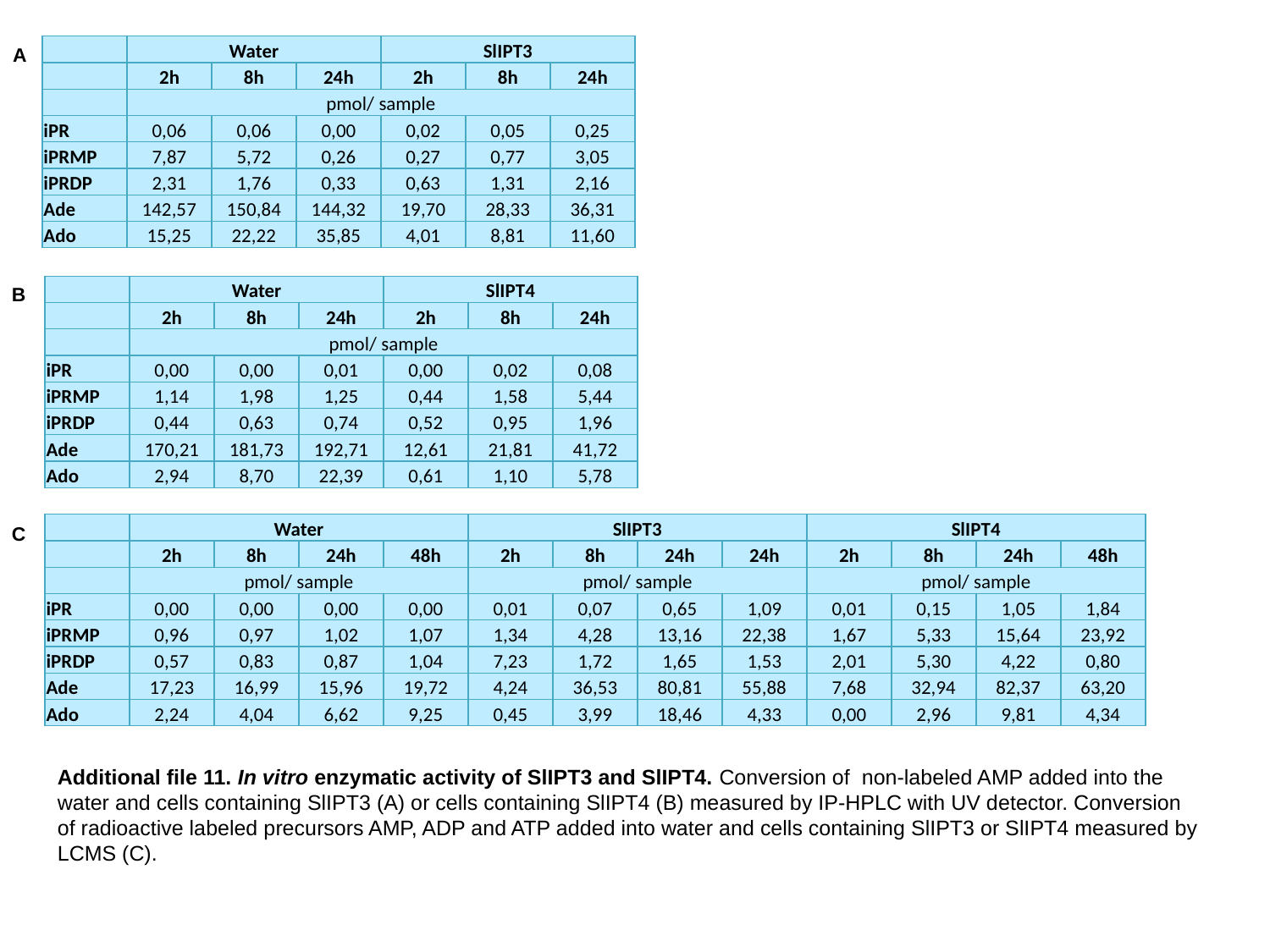

A
| | Water | | | SlIPT3 | | |
| --- | --- | --- | --- | --- | --- | --- |
| | 2h | 8h | 24h | 2h | 8h | 24h |
| | pmol/ sample | | | | | |
| iPR | 0,06 | 0,06 | 0,00 | 0,02 | 0,05 | 0,25 |
| iPRMP | 7,87 | 5,72 | 0,26 | 0,27 | 0,77 | 3,05 |
| iPRDP | 2,31 | 1,76 | 0,33 | 0,63 | 1,31 | 2,16 |
| Ade | 142,57 | 150,84 | 144,32 | 19,70 | 28,33 | 36,31 |
| Ado | 15,25 | 22,22 | 35,85 | 4,01 | 8,81 | 11,60 |
B
| | Water | | | SlIPT4 | | |
| --- | --- | --- | --- | --- | --- | --- |
| | 2h | 8h | 24h | 2h | 8h | 24h |
| | pmol/ sample | | | | | |
| iPR | 0,00 | 0,00 | 0,01 | 0,00 | 0,02 | 0,08 |
| iPRMP | 1,14 | 1,98 | 1,25 | 0,44 | 1,58 | 5,44 |
| iPRDP | 0,44 | 0,63 | 0,74 | 0,52 | 0,95 | 1,96 |
| Ade | 170,21 | 181,73 | 192,71 | 12,61 | 21,81 | 41,72 |
| Ado | 2,94 | 8,70 | 22,39 | 0,61 | 1,10 | 5,78 |
| | Water | | | | SlIPT3 | | | | SlIPT4 | | | |
| --- | --- | --- | --- | --- | --- | --- | --- | --- | --- | --- | --- | --- |
| | 2h | 8h | 24h | 48h | 2h | 8h | 24h | 24h | 2h | 8h | 24h | 48h |
| | pmol/ sample | | | | pmol/ sample | | | | pmol/ sample | | | |
| iPR | 0,00 | 0,00 | 0,00 | 0,00 | 0,01 | 0,07 | 0,65 | 1,09 | 0,01 | 0,15 | 1,05 | 1,84 |
| iPRMP | 0,96 | 0,97 | 1,02 | 1,07 | 1,34 | 4,28 | 13,16 | 22,38 | 1,67 | 5,33 | 15,64 | 23,92 |
| iPRDP | 0,57 | 0,83 | 0,87 | 1,04 | 7,23 | 1,72 | 1,65 | 1,53 | 2,01 | 5,30 | 4,22 | 0,80 |
| Ade | 17,23 | 16,99 | 15,96 | 19,72 | 4,24 | 36,53 | 80,81 | 55,88 | 7,68 | 32,94 | 82,37 | 63,20 |
| Ado | 2,24 | 4,04 | 6,62 | 9,25 | 0,45 | 3,99 | 18,46 | 4,33 | 0,00 | 2,96 | 9,81 | 4,34 |
C
Additional file 11. In vitro enzymatic activity of SlIPT3 and SlIPT4. Conversion of non-labeled AMP added into the water and cells containing SlIPT3 (A) or cells containing SlIPT4 (B) measured by IP-HPLC with UV detector. Conversion of radioactive labeled precursors AMP, ADP and ATP added into water and cells containing SlIPT3 or SlIPT4 measured by LCMS (C).
