# Supplementary material for: Tomato (Solanum lycopersicum L.) SlIPT3 and SlIPT4 isopentenyltransferases mediate salt stress response in tomato
Source: BMC Plant Biol. 2015 Mar 12;15:85. doi: 10.1186/s12870-015-0415-7 (PMC4404076; doi:10.1186/s12870-015-0415-7)
Supplement: Additional file 4: — Phenotype of T1-generation tomatoes 35::SlIPT3 Line 1 (L1) transformants. [file 12870_2015_415_MOESM4_ESM.pptx]

## Slide 1
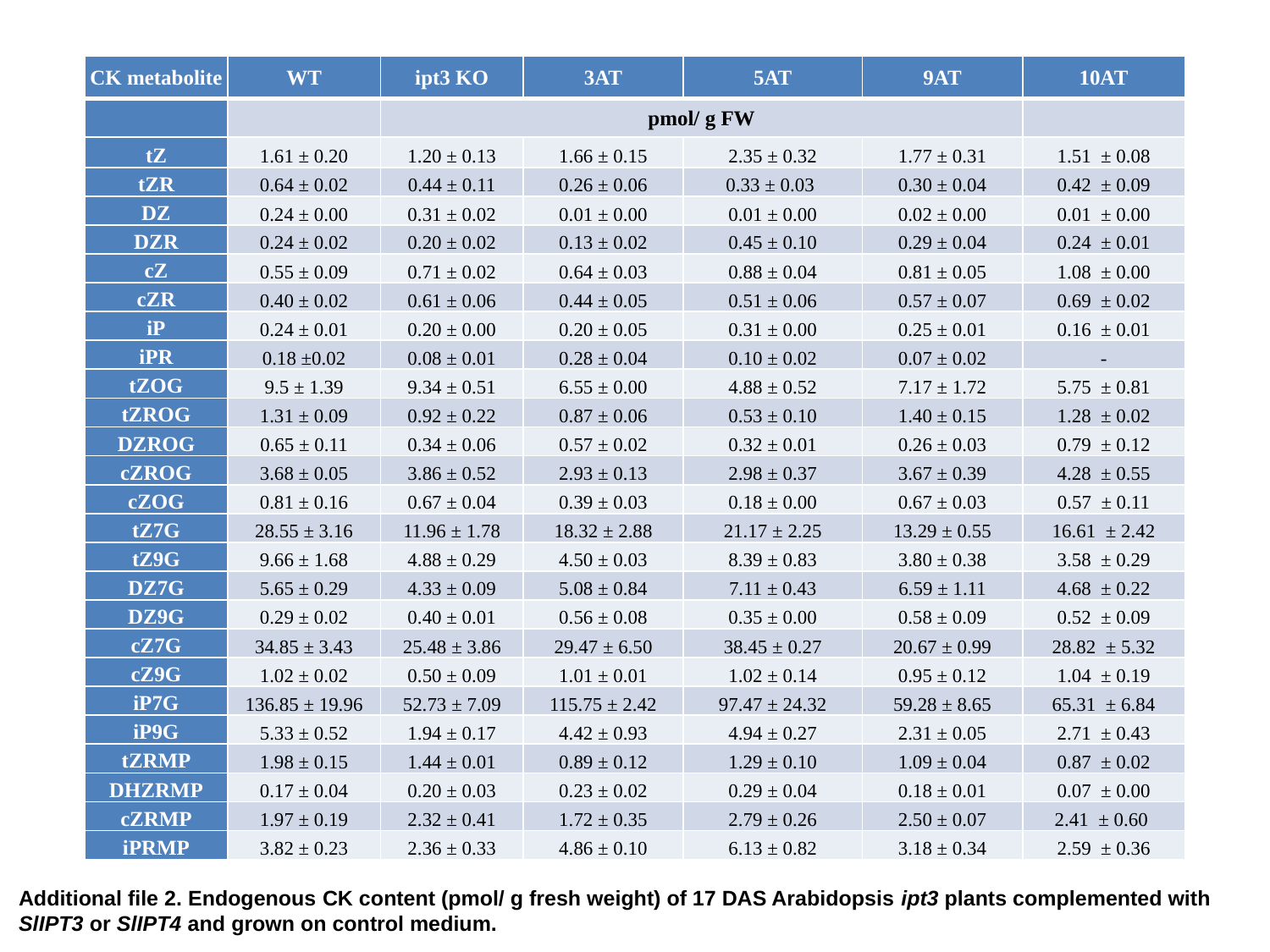

| CK metabolite | WT | ipt3 KO | 3AT | 5AT | 9AT | 10AT |
| --- | --- | --- | --- | --- | --- | --- |
| | | pmol/ g FW | | | | |
| tZ | 1.61 ± 0.20 | 1.20 ± 0.13 | 1.66 ± 0.15 | 2.35 ± 0.32 | 1.77 ± 0.31 | 1.51 ± 0.08 |
| tZR | 0.64 ± 0.02 | 0.44 ± 0.11 | 0.26 ± 0.06 | 0.33 ± 0.03 | 0.30 ± 0.04 | 0.42 ± 0.09 |
| DZ | 0.24 ± 0.00 | 0.31 ± 0.02 | 0.01 ± 0.00 | 0.01 ± 0.00 | 0.02 ± 0.00 | 0.01 ± 0.00 |
| DZR | 0.24 ± 0.02 | 0.20 ± 0.02 | 0.13 ± 0.02 | 0.45 ± 0.10 | 0.29 ± 0.04 | 0.24 ± 0.01 |
| cZ | 0.55 ± 0.09 | 0.71 ± 0.02 | 0.64 ± 0.03 | 0.88 ± 0.04 | 0.81 ± 0.05 | 1.08 ± 0.00 |
| cZR | 0.40 ± 0.02 | 0.61 ± 0.06 | 0.44 ± 0.05 | 0.51 ± 0.06 | 0.57 ± 0.07 | 0.69 ± 0.02 |
| iP | 0.24 ± 0.01 | 0.20 ± 0.00 | 0.20 ± 0.05 | 0.31 ± 0.00 | 0.25 ± 0.01 | 0.16 ± 0.01 |
| iPR | 0.18 ±0.02 | 0.08 ± 0.01 | 0.28 ± 0.04 | 0.10 ± 0.02 | 0.07 ± 0.02 | - |
| tZOG | 9.5 ± 1.39 | 9.34 ± 0.51 | 6.55 ± 0.00 | 4.88 ± 0.52 | 7.17 ± 1.72 | 5.75 ± 0.81 |
| tZROG | 1.31 ± 0.09 | 0.92 ± 0.22 | 0.87 ± 0.06 | 0.53 ± 0.10 | 1.40 ± 0.15 | 1.28 ± 0.02 |
| DZROG | 0.65 ± 0.11 | 0.34 ± 0.06 | 0.57 ± 0.02 | 0.32 ± 0.01 | 0.26 ± 0.03 | 0.79 ± 0.12 |
| cZROG | 3.68 ± 0.05 | 3.86 ± 0.52 | 2.93 ± 0.13 | 2.98 ± 0.37 | 3.67 ± 0.39 | 4.28 ± 0.55 |
| cZOG | 0.81 ± 0.16 | 0.67 ± 0.04 | 0.39 ± 0.03 | 0.18 ± 0.00 | 0.67 ± 0.03 | 0.57 ± 0.11 |
| tZ7G | 28.55 ± 3.16 | 11.96 ± 1.78 | 18.32 ± 2.88 | 21.17 ± 2.25 | 13.29 ± 0.55 | 16.61 ± 2.42 |
| tZ9G | 9.66 ± 1.68 | 4.88 ± 0.29 | 4.50 ± 0.03 | 8.39 ± 0.83 | 3.80 ± 0.38 | 3.58 ± 0.29 |
| DZ7G | 5.65 ± 0.29 | 4.33 ± 0.09 | 5.08 ± 0.84 | 7.11 ± 0.43 | 6.59 ± 1.11 | 4.68 ± 0.22 |
| DZ9G | 0.29 ± 0.02 | 0.40 ± 0.01 | 0.56 ± 0.08 | 0.35 ± 0.00 | 0.58 ± 0.09 | 0.52 ± 0.09 |
| cZ7G | 34.85 ± 3.43 | 25.48 ± 3.86 | 29.47 ± 6.50 | 38.45 ± 0.27 | 20.67 ± 0.99 | 28.82 ± 5.32 |
| cZ9G | 1.02 ± 0.02 | 0.50 ± 0.09 | 1.01 ± 0.01 | 1.02 ± 0.14 | 0.95 ± 0.12 | 1.04 ± 0.19 |
| iP7G | 136.85 ± 19.96 | 52.73 ± 7.09 | 115.75 ± 2.42 | 97.47 ± 24.32 | 59.28 ± 8.65 | 65.31 ± 6.84 |
| iP9G | 5.33 ± 0.52 | 1.94 ± 0.17 | 4.42 ± 0.93 | 4.94 ± 0.27 | 2.31 ± 0.05 | 2.71 ± 0.43 |
| tZRMP | 1.98 ± 0.15 | 1.44 ± 0.01 | 0.89 ± 0.12 | 1.29 ± 0.10 | 1.09 ± 0.04 | 0.87 ± 0.02 |
| DHZRMP | 0.17 ± 0.04 | 0.20 ± 0.03 | 0.23 ± 0.02 | 0.29 ± 0.04 | 0.18 ± 0.01 | 0.07 ± 0.00 |
| cZRMP | 1.97 ± 0.19 | 2.32 ± 0.41 | 1.72 ± 0.35 | 2.79 ± 0.26 | 2.50 ± 0.07 | 2.41 ± 0.60 |
| iPRMP | 3.82 ± 0.23 | 2.36 ± 0.33 | 4.86 ± 0.10 | 6.13 ± 0.82 | 3.18 ± 0.34 | 2.59 ± 0.36 |
Additional file 2. Endogenous CK content (pmol/ g fresh weight) of 17 DAS Arabidopsis ipt3 plants complemented with SlIPT3 or SlIPT4 and grown on control medium.
