# Supplementary material for: Tomato (Solanum lycopersicum L.) SlIPT3 and SlIPT4 isopentenyltransferases mediate salt stress response in tomato
Source: BMC Plant Biol. 2015 Mar 12;15:85. doi: 10.1186/s12870-015-0415-7 (PMC4404076; doi:10.1186/s12870-015-0415-7)
Supplement: Additional file 5: — Endogenous CKs content (pmol/g FW) in young leaves of T0 35S::SlIPT3 tomatoes. The system of abbreviations was adopted and modified according to published reference [60]. [file 12870_2015_415_MOESM5_ESM.pptx]

## Slide 1
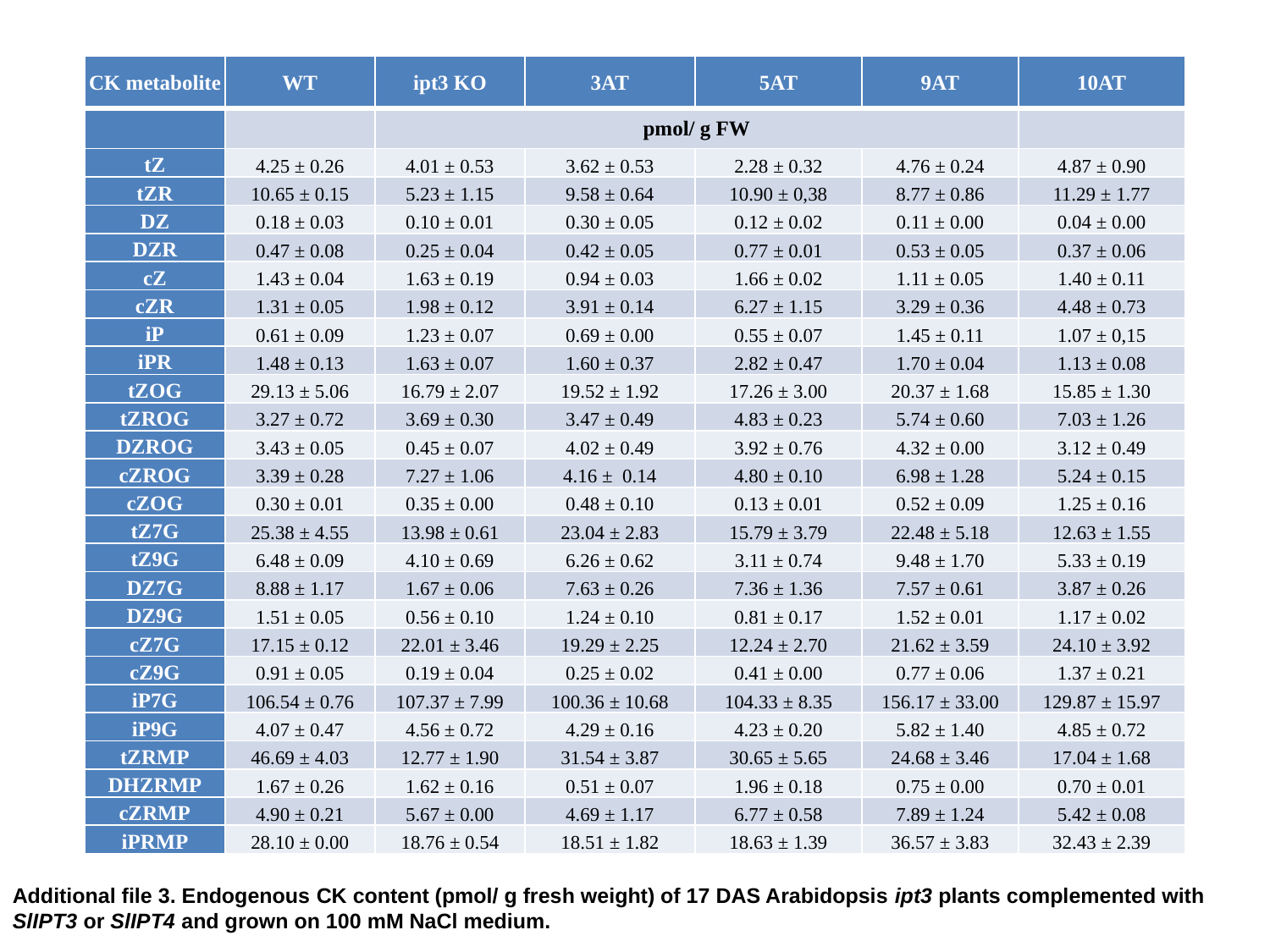

| CK metabolite | WT | ipt3 KO | 3AT | 5AT | 9AT | 10AT |
| --- | --- | --- | --- | --- | --- | --- |
| | | pmol/ g FW | | | | |
| tZ | 4.25 ± 0.26 | 4.01 ± 0.53 | 3.62 ± 0.53 | 2.28 ± 0.32 | 4.76 ± 0.24 | 4.87 ± 0.90 |
| tZR | 10.65 ± 0.15 | 5.23 ± 1.15 | 9.58 ± 0.64 | 10.90 ± 0,38 | 8.77 ± 0.86 | 11.29 ± 1.77 |
| DZ | 0.18 ± 0.03 | 0.10 ± 0.01 | 0.30 ± 0.05 | 0.12 ± 0.02 | 0.11 ± 0.00 | 0.04 ± 0.00 |
| DZR | 0.47 ± 0.08 | 0.25 ± 0.04 | 0.42 ± 0.05 | 0.77 ± 0.01 | 0.53 ± 0.05 | 0.37 ± 0.06 |
| cZ | 1.43 ± 0.04 | 1.63 ± 0.19 | 0.94 ± 0.03 | 1.66 ± 0.02 | 1.11 ± 0.05 | 1.40 ± 0.11 |
| cZR | 1.31 ± 0.05 | 1.98 ± 0.12 | 3.91 ± 0.14 | 6.27 ± 1.15 | 3.29 ± 0.36 | 4.48 ± 0.73 |
| iP | 0.61 ± 0.09 | 1.23 ± 0.07 | 0.69 ± 0.00 | 0.55 ± 0.07 | 1.45 ± 0.11 | 1.07 ± 0,15 |
| iPR | 1.48 ± 0.13 | 1.63 ± 0.07 | 1.60 ± 0.37 | 2.82 ± 0.47 | 1.70 ± 0.04 | 1.13 ± 0.08 |
| tZOG | 29.13 ± 5.06 | 16.79 ± 2.07 | 19.52 ± 1.92 | 17.26 ± 3.00 | 20.37 ± 1.68 | 15.85 ± 1.30 |
| tZROG | 3.27 ± 0.72 | 3.69 ± 0.30 | 3.47 ± 0.49 | 4.83 ± 0.23 | 5.74 ± 0.60 | 7.03 ± 1.26 |
| DZROG | 3.43 ± 0.05 | 0.45 ± 0.07 | 4.02 ± 0.49 | 3.92 ± 0.76 | 4.32 ± 0.00 | 3.12 ± 0.49 |
| cZROG | 3.39 ± 0.28 | 7.27 ± 1.06 | 4.16 ± 0.14 | 4.80 ± 0.10 | 6.98 ± 1.28 | 5.24 ± 0.15 |
| cZOG | 0.30 ± 0.01 | 0.35 ± 0.00 | 0.48 ± 0.10 | 0.13 ± 0.01 | 0.52 ± 0.09 | 1.25 ± 0.16 |
| tZ7G | 25.38 ± 4.55 | 13.98 ± 0.61 | 23.04 ± 2.83 | 15.79 ± 3.79 | 22.48 ± 5.18 | 12.63 ± 1.55 |
| tZ9G | 6.48 ± 0.09 | 4.10 ± 0.69 | 6.26 ± 0.62 | 3.11 ± 0.74 | 9.48 ± 1.70 | 5.33 ± 0.19 |
| DZ7G | 8.88 ± 1.17 | 1.67 ± 0.06 | 7.63 ± 0.26 | 7.36 ± 1.36 | 7.57 ± 0.61 | 3.87 ± 0.26 |
| DZ9G | 1.51 ± 0.05 | 0.56 ± 0.10 | 1.24 ± 0.10 | 0.81 ± 0.17 | 1.52 ± 0.01 | 1.17 ± 0.02 |
| cZ7G | 17.15 ± 0.12 | 22.01 ± 3.46 | 19.29 ± 2.25 | 12.24 ± 2.70 | 21.62 ± 3.59 | 24.10 ± 3.92 |
| cZ9G | 0.91 ± 0.05 | 0.19 ± 0.04 | 0.25 ± 0.02 | 0.41 ± 0.00 | 0.77 ± 0.06 | 1.37 ± 0.21 |
| iP7G | 106.54 ± 0.76 | 107.37 ± 7.99 | 100.36 ± 10.68 | 104.33 ± 8.35 | 156.17 ± 33.00 | 129.87 ± 15.97 |
| iP9G | 4.07 ± 0.47 | 4.56 ± 0.72 | 4.29 ± 0.16 | 4.23 ± 0.20 | 5.82 ± 1.40 | 4.85 ± 0.72 |
| tZRMP | 46.69 ± 4.03 | 12.77 ± 1.90 | 31.54 ± 3.87 | 30.65 ± 5.65 | 24.68 ± 3.46 | 17.04 ± 1.68 |
| DHZRMP | 1.67 ± 0.26 | 1.62 ± 0.16 | 0.51 ± 0.07 | 1.96 ± 0.18 | 0.75 ± 0.00 | 0.70 ± 0.01 |
| cZRMP | 4.90 ± 0.21 | 5.67 ± 0.00 | 4.69 ± 1.17 | 6.77 ± 0.58 | 7.89 ± 1.24 | 5.42 ± 0.08 |
| iPRMP | 28.10 ± 0.00 | 18.76 ± 0.54 | 18.51 ± 1.82 | 18.63 ± 1.39 | 36.57 ± 3.83 | 32.43 ± 2.39 |
Additional file 3. Endogenous CK content (pmol/ g fresh weight) of 17 DAS Arabidopsis ipt3 plants complemented with SlIPT3 or SlIPT4 and grown on 100 mM NaCl medium.
