# Supplementary figures and images for: Tomato (Solanum lycopersicum L.) SlIPT3 and SlIPT4 isopentenyltransferases mediate salt stress response in tomato
Source: BMC Plant Biol. 2015 Mar 12;15:85. doi: 10.1186/s12870-015-0415-7 (PMC4404076; doi:10.1186/s12870-015-0415-7)

## Slide 1
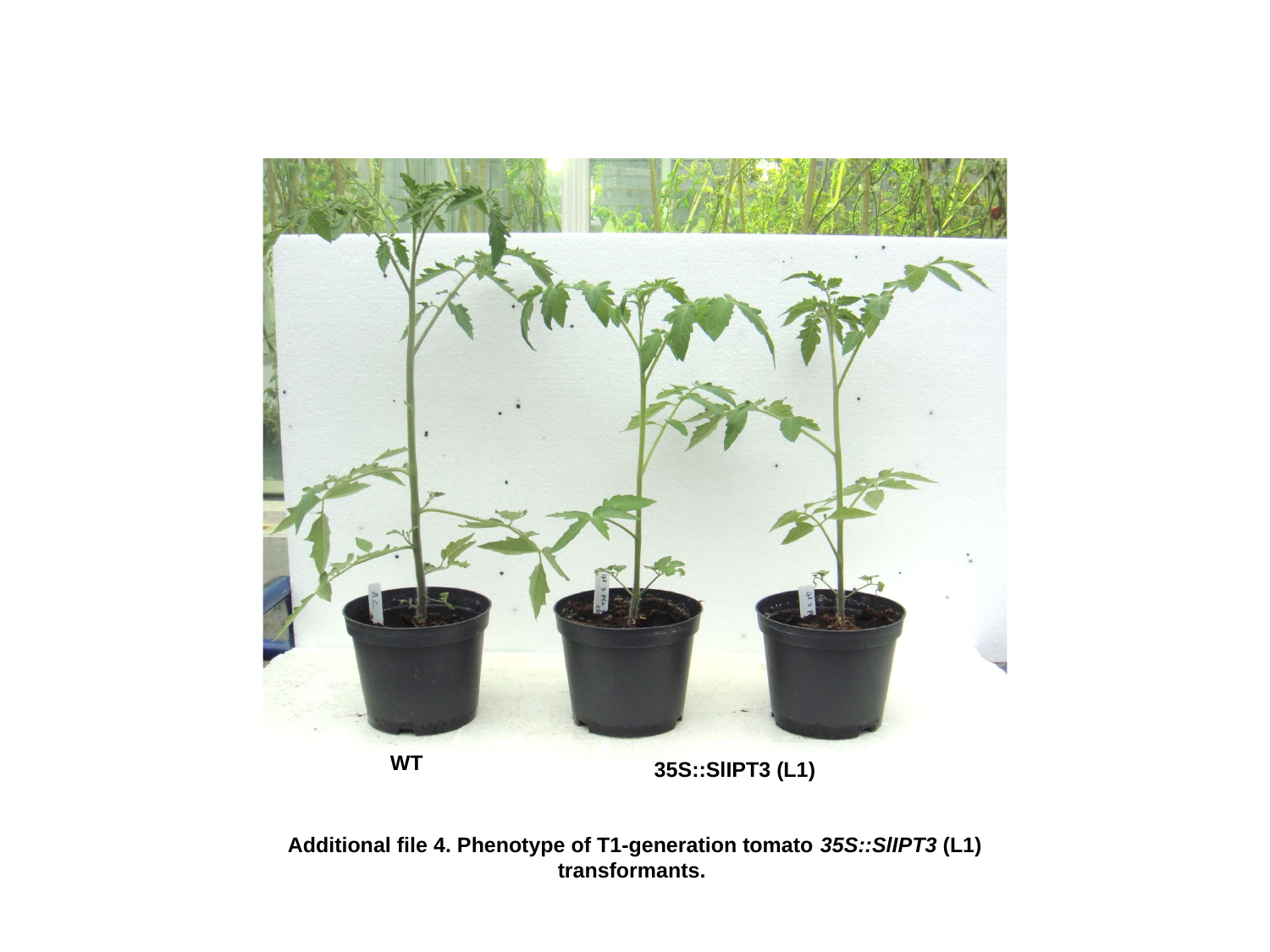

WT
35S::SlIPT3 (L1)
Additional file 4. Phenotype of T1-generation tomato 35S::SlIPT3 (L1) transformants.

Supplement: Additional file 6: — Cytokinin oxidase/dehydrogenase (CKX) activity in 35S::SlIPT3 tomato young leaves (L6 and L7). [file 12870_2015_415_MOESM6_ESM.pptx]

## Slide 1
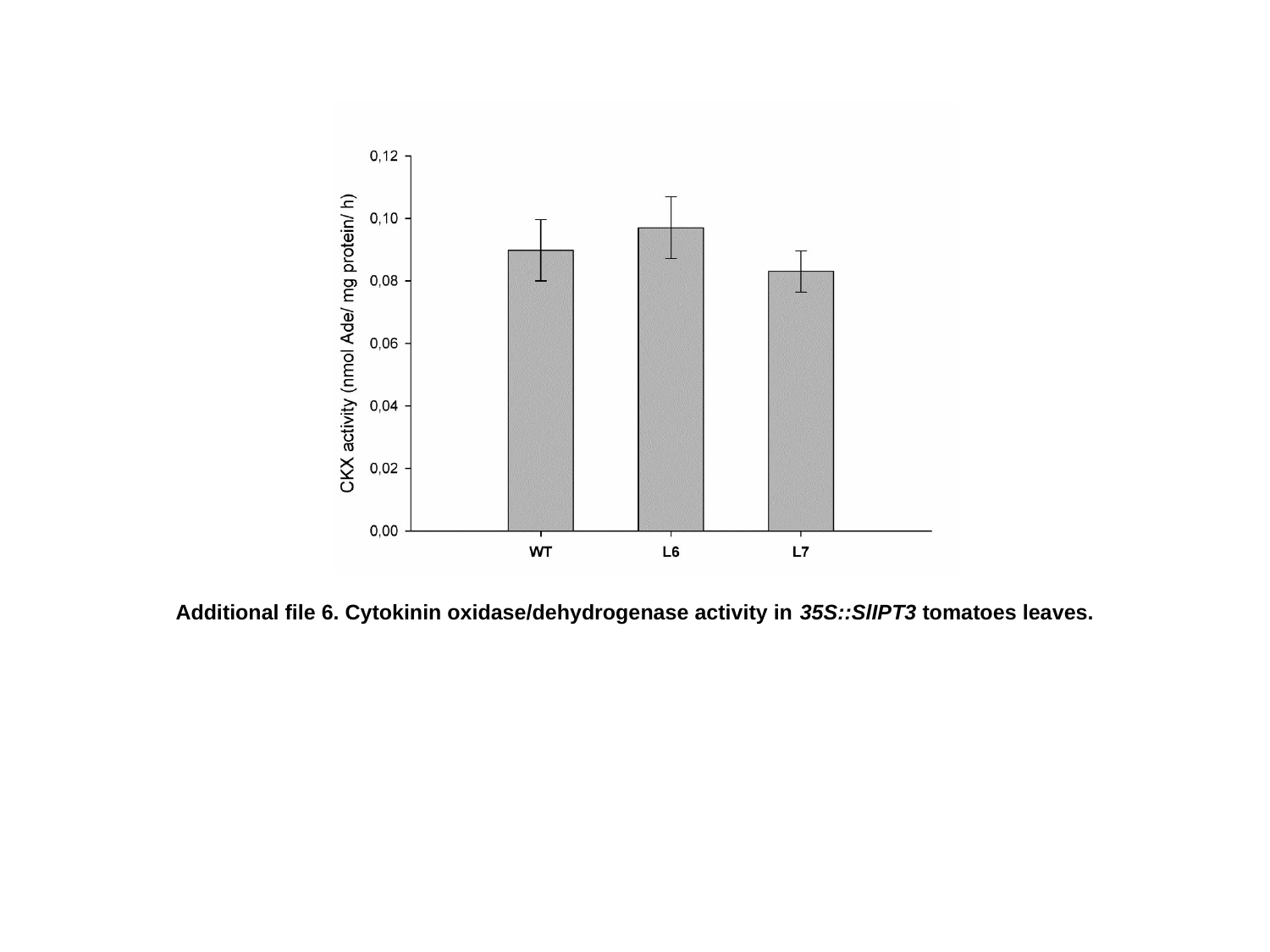

Additional file 6. Cytokinin oxidase/dehydrogenase activity in 35S::SlIPT3 tomatoes leaves.

Supplement: Additional file 8: — Hypothetical scheme of SlIPT3 and SlIPT4 response to early salt stress and the feedback regulation by exogenous CK treatment in tomato plants. After salt stress (150 mM NaCl) treatment, immediate down-regulation of SlIPT3 and SlIPT4 transcripts with a subsequent up-regulation in tomato vegetative organs was determined. Repression of both genes after exogenous application of tZ demonstrated the complexity of CK networks, while the overabundance of CKs (specially iP7G) in 35S::SlIPT3 may ensure a stronger phenotype under salt stress (100 mM NaCl) conditions. [file 12870_2015_415_MOESM8_ESM.pptx]
