# Supplementary material for: Tomato (Solanum lycopersicum L.) SlIPT3 and SlIPT4 isopentenyltransferases mediate salt stress response in tomato
Source: BMC Plant Biol. 2015 Mar 12;15:85. doi: 10.1186/s12870-015-0415-7 (PMC4404076; doi:10.1186/s12870-015-0415-7)
Supplement: Additional file 7: — The content of photosynthetic pigments (μg/g DW) in 4 weeks-old tomato developing leaves of WT and T2-generation of 35S::SlIPT3 (L1) tomato plants cultivated on the control and salt growth media. DEPS (De-epoxidation state) is calculated according to formula: DEPS = (0.5*A + Z)/(V + A + Z); DEPS (De-epoxidation state per chlorophyll) is calculated according to formula: DEPSC = (0.5*A + Z)/(Chl a + b). ^ expressed in relative unit. *Data represent means and SD of two replicates. *significantly different datasets from WT (unpaired Student's t-test, P ≤ 0.05). [file 12870_2015_415_MOESM7_ESM.pptx]

## Slide 1
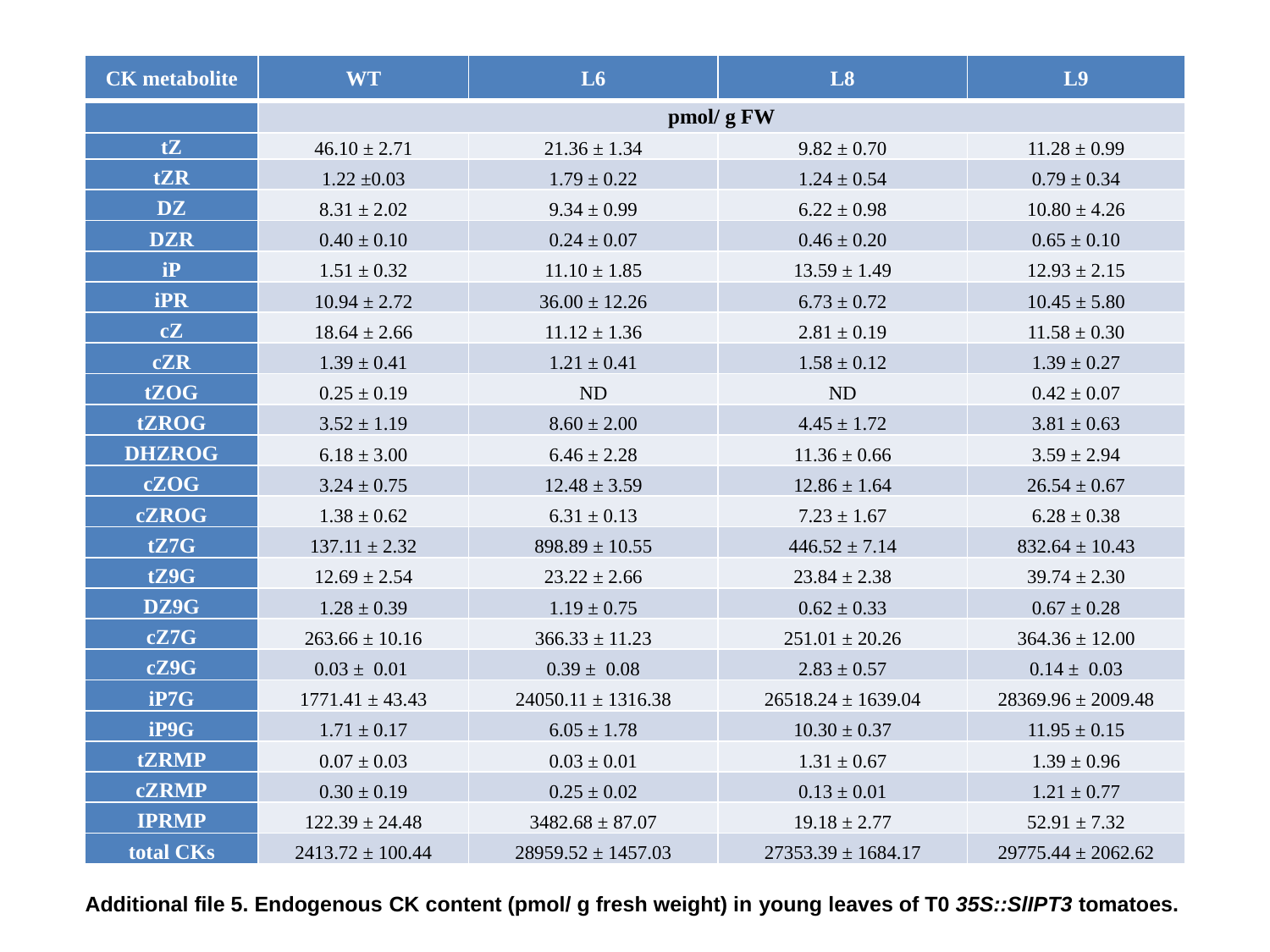

| CK metabolite | WT | L6 | L8 | L9 |
| --- | --- | --- | --- | --- |
| | pmol/ g FW | | | |
| tZ | 46.10 ± 2.71 | 21.36 ± 1.34 | 9.82 ± 0.70 | 11.28 ± 0.99 |
| tZR | 1.22 ±0.03 | 1.79 ± 0.22 | 1.24 ± 0.54 | 0.79 ± 0.34 |
| DZ | 8.31 ± 2.02 | 9.34 ± 0.99 | 6.22 ± 0.98 | 10.80 ± 4.26 |
| DZR | 0.40 ± 0.10 | 0.24 ± 0.07 | 0.46 ± 0.20 | 0.65 ± 0.10 |
| iP | 1.51 ± 0.32 | 11.10 ± 1.85 | 13.59 ± 1.49 | 12.93 ± 2.15 |
| iPR | 10.94 ± 2.72 | 36.00 ± 12.26 | 6.73 ± 0.72 | 10.45 ± 5.80 |
| cZ | 18.64 ± 2.66 | 11.12 ± 1.36 | 2.81 ± 0.19 | 11.58 ± 0.30 |
| cZR | 1.39 ± 0.41 | 1.21 ± 0.41 | 1.58 ± 0.12 | 1.39 ± 0.27 |
| tZOG | 0.25 ± 0.19 | ND | ND | 0.42 ± 0.07 |
| tZROG | 3.52 ± 1.19 | 8.60 ± 2.00 | 4.45 ± 1.72 | 3.81 ± 0.63 |
| DHZROG | 6.18 ± 3.00 | 6.46 ± 2.28 | 11.36 ± 0.66 | 3.59 ± 2.94 |
| cZOG | 3.24 ± 0.75 | 12.48 ± 3.59 | 12.86 ± 1.64 | 26.54 ± 0.67 |
| cZROG | 1.38 ± 0.62 | 6.31 ± 0.13 | 7.23 ± 1.67 | 6.28 ± 0.38 |
| tZ7G | 137.11 ± 2.32 | 898.89 ± 10.55 | 446.52 ± 7.14 | 832.64 ± 10.43 |
| tZ9G | 12.69 ± 2.54 | 23.22 ± 2.66 | 23.84 ± 2.38 | 39.74 ± 2.30 |
| DZ9G | 1.28 ± 0.39 | 1.19 ± 0.75 | 0.62 ± 0.33 | 0.67 ± 0.28 |
| cZ7G | 263.66 ± 10.16 | 366.33 ± 11.23 | 251.01 ± 20.26 | 364.36 ± 12.00 |
| cZ9G | 0.03 ± 0.01 | 0.39 ± 0.08 | 2.83 ± 0.57 | 0.14 ± 0.03 |
| iP7G | 1771.41 ± 43.43 | 24050.11 ± 1316.38 | 26518.24 ± 1639.04 | 28369.96 ± 2009.48 |
| iP9G | 1.71 ± 0.17 | 6.05 ± 1.78 | 10.30 ± 0.37 | 11.95 ± 0.15 |
| tZRMP | 0.07 ± 0.03 | 0.03 ± 0.01 | 1.31 ± 0.67 | 1.39 ± 0.96 |
| cZRMP | 0.30 ± 0.19 | 0.25 ± 0.02 | 0.13 ± 0.01 | 1.21 ± 0.77 |
| IPRMP | 122.39 ± 24.48 | 3482.68 ± 87.07 | 19.18 ± 2.77 | 52.91 ± 7.32 |
| total CKs | 2413.72 ± 100.44 | 28959.52 ± 1457.03 | 27353.39 ± 1684.17 | 29775.44 ± 2062.62 |
Additional file 5. Endogenous CK content (pmol/ g fresh weight) in young leaves of T0 35S::SlIPT3 tomatoes.
