# Supplementary material for: Tomato (Solanum lycopersicum L.) SlIPT3 and SlIPT4 isopentenyltransferases mediate salt stress response in tomato
Source: BMC Plant Biol. 2015 Mar 12;15:85. doi: 10.1186/s12870-015-0415-7 (PMC4404076; doi:10.1186/s12870-015-0415-7)
Supplement: Additional file 9: — Sequences of tomato primers used for qRT-PCR analysis. [file 12870_2015_415_MOESM9_ESM.pptx]

## Slide 1
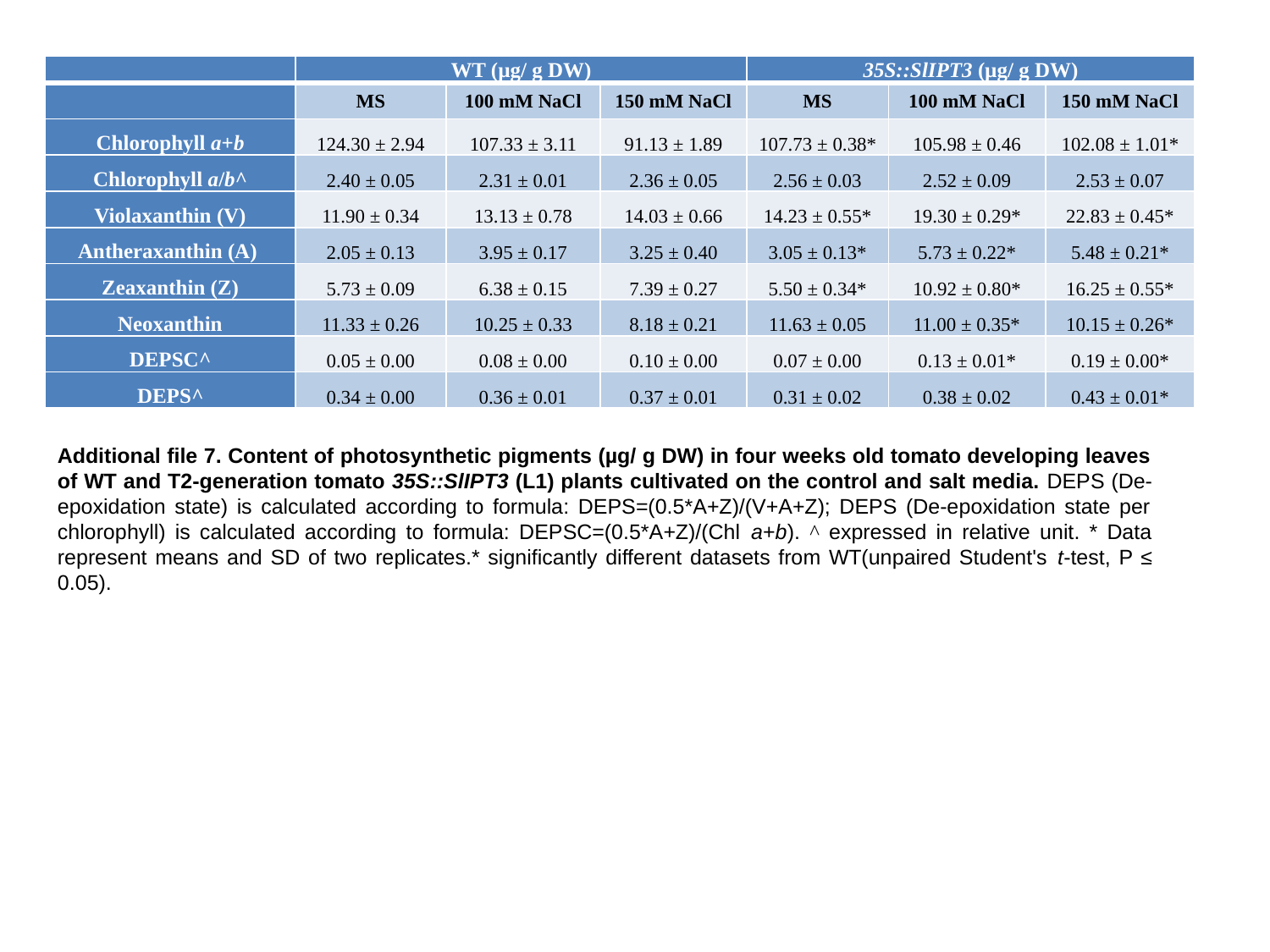

| | WT (µg/ g DW) | | | 35S::SlIPT3 (µg/ g DW) | | |
| --- | --- | --- | --- | --- | --- | --- |
| | MS | 100 mM NaCl | 150 mM NaCl | MS | 100 mM NaCl | 150 mM NaCl |
| Chlorophyll a+b | 124.30 ± 2.94 | 107.33 ± 3.11 | 91.13 ± 1.89 | 107.73 ± 0.38\* | 105.98 ± 0.46 | 102.08 ± 1.01\* |
| Chlorophyll a/b^ | 2.40 ± 0.05 | 2.31 ± 0.01 | 2.36 ± 0.05 | 2.56 ± 0.03 | 2.52 ± 0.09 | 2.53 ± 0.07 |
| Violaxanthin (V) | 11.90 ± 0.34 | 13.13 ± 0.78 | 14.03 ± 0.66 | 14.23 ± 0.55\* | 19.30 ± 0.29\* | 22.83 ± 0.45\* |
| Antheraxanthin (A) | 2.05 ± 0.13 | 3.95 ± 0.17 | 3.25 ± 0.40 | 3.05 ± 0.13\* | 5.73 ± 0.22\* | 5.48 ± 0.21\* |
| Zeaxanthin (Z) | 5.73 ± 0.09 | 6.38 ± 0.15 | 7.39 ± 0.27 | 5.50 ± 0.34\* | 10.92 ± 0.80\* | 16.25 ± 0.55\* |
| Neoxanthin | 11.33 ± 0.26 | 10.25 ± 0.33 | 8.18 ± 0.21 | 11.63 ± 0.05 | 11.00 ± 0.35\* | 10.15 ± 0.26\* |
| DEPSC^ | 0.05 ± 0.00 | 0.08 ± 0.00 | 0.10 ± 0.00 | 0.07 ± 0.00 | 0.13 ± 0.01\* | 0.19 ± 0.00\* |
| DEPS^ | 0.34 ± 0.00 | 0.36 ± 0.01 | 0.37 ± 0.01 | 0.31 ± 0.02 | 0.38 ± 0.02 | 0.43 ± 0.01\* |
Additional file 7. Content of photosynthetic pigments (µg/ g DW) in four weeks old tomato developing leaves of WT and T2-generation tomato 35S::SlIPT3 (L1) plants cultivated on the control and salt media. DEPS (De-epoxidation state) is calculated according to formula: DEPS=(0.5*A+Z)/(V+A+Z); DEPS (De-epoxidation state per chlorophyll) is calculated according to formula: DEPSC=(0.5*A+Z)/(Chl a+b). ^ expressed in relative unit. * Data represent means and SD of two replicates.* significantly different datasets from WT(unpaired Student's t-test, P ≤ 0.05).
