# Supplementary material for: Tomato (Solanum lycopersicum L.) SlIPT3 and SlIPT4 isopentenyltransferases mediate salt stress response in tomato
Source: BMC Plant Biol. 2015 Mar 12;15:85. doi: 10.1186/s12870-015-0415-7 (PMC4404076; doi:10.1186/s12870-015-0415-7)
Supplement: Additional file 10: — Sequences of Arabidopsis primers used for qRT-PCR analysis. [file 12870_2015_415_MOESM10_ESM.pptx]

## Slide 1
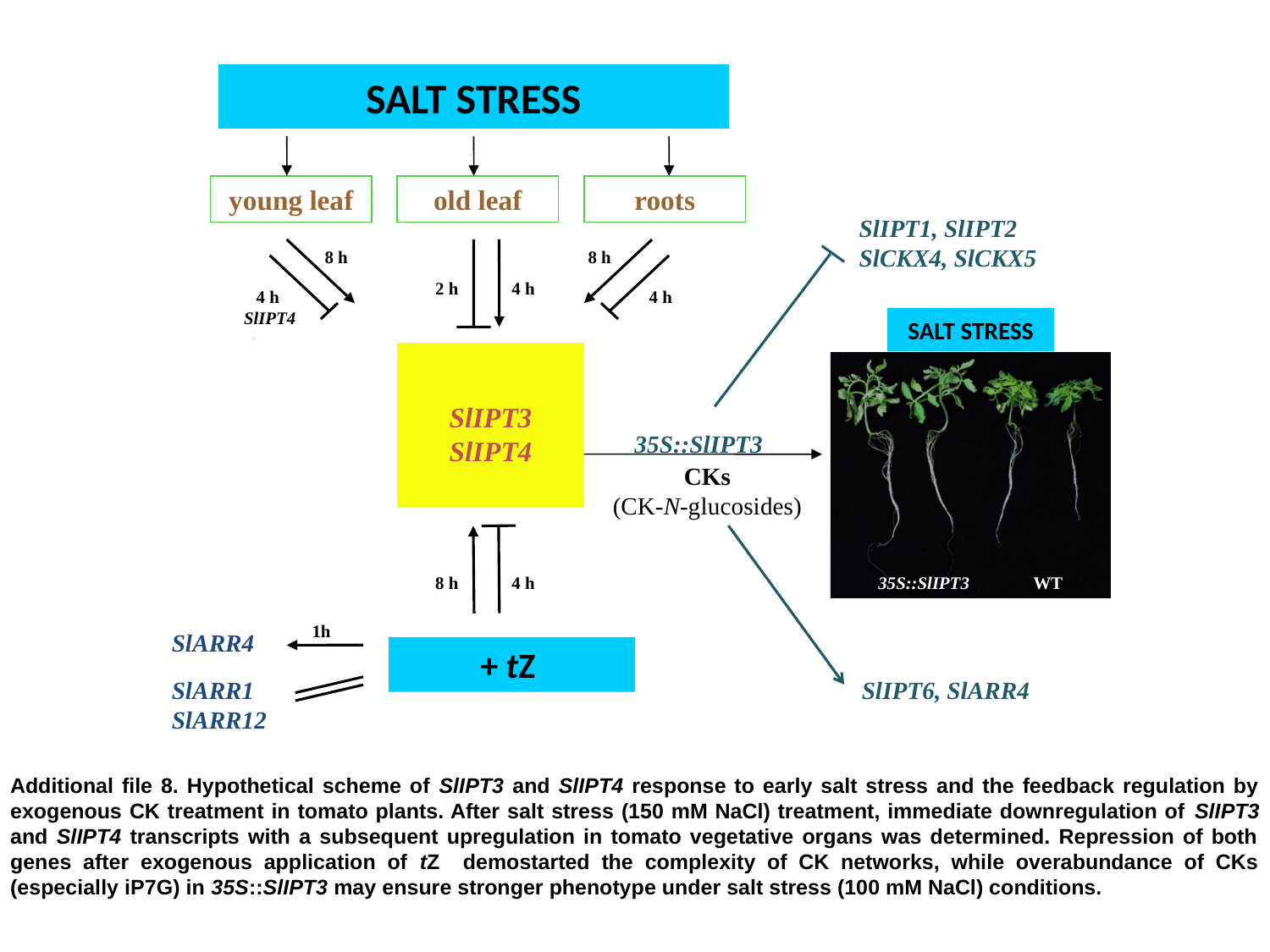

SALT STRESS
young leaf
old leaf
roots
SlIPT1, SlIPT2
SlCKX4, SlCKX5
8 h
4 h
SlIPT4
2 h
4 h
8 h
4 h
SlIPT3
SlIPT4
8 h
4 h
1h
SlARR4
SlARR1
SlARR12
+ tZ
SALT STRESS
35S::SlIPT3
WT
35S::SlIPT3
CKs
(CK-N-glucosides)
SlIPT6, SlARR4
Additional file 8. Hypothetical scheme of SlIPT3 and SlIPT4 response to early salt stress and the feedback regulation by exogenous CK treatment in tomato plants. After salt stress (150 mM NaCl) treatment, immediate downregulation of SlIPT3 and SlIPT4 transcripts with a subsequent upregulation in tomato vegetative organs was determined. Repression of both genes after exogenous application of tZ demostarted the complexity of CK networks, while overabundance of CKs (especially iP7G) in 35S::SlIPT3 may ensure stronger phenotype under salt stress (100 mM NaCl) conditions.
