# Supplementary material for: Tomato (Solanum lycopersicum L.) SlIPT3 and SlIPT4 isopentenyltransferases mediate salt stress response in tomato
Source: BMC Plant Biol. 2015 Mar 12;15:85. doi: 10.1186/s12870-015-0415-7 (PMC4404076; doi:10.1186/s12870-015-0415-7)
Supplement: Additional file 11: — In vitro enzymatic activity of SlIPT3 and SlIPT4. Conversion of non-labeled AMP added into the water and cells containing SlIPT3 (A) or cells containing SlIPT4 (B) measured by IP-HPLC with UV detector. Conversion of radioactive labeled precursors AMP, ADP and ATP added into the water and cells containing SlIPT3 or SlIPT4 were measured by LCMS (C). [file 12870_2015_415_MOESM11_ESM.pptx]

## Slide 1
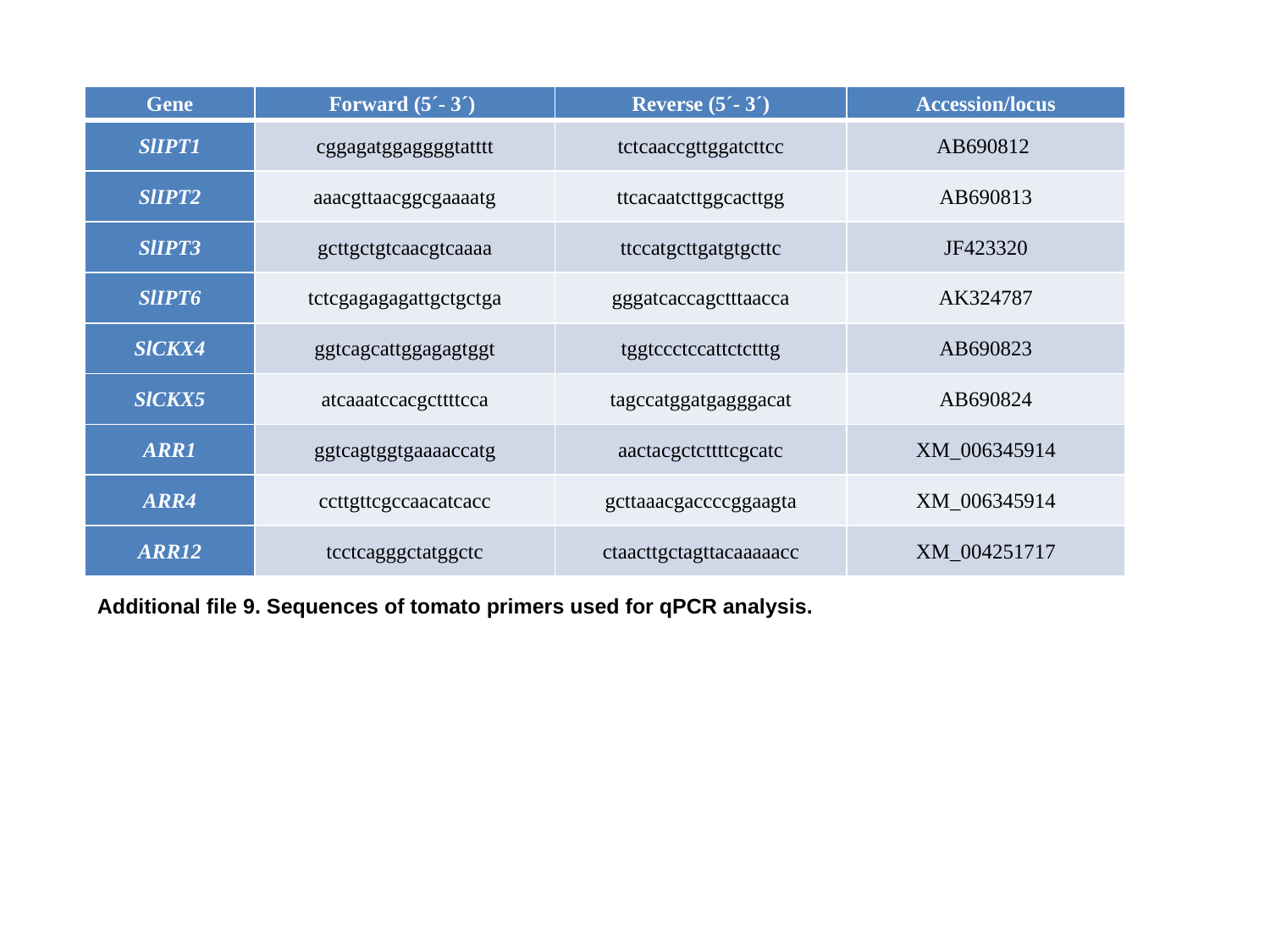

| Gene | Forward (5´- 3´) | Reverse (5´- 3´) | Accession/locus |
| --- | --- | --- | --- |
| SlIPT1 | cggagatggaggggtatttt | tctcaaccgttggatcttcc | AB690812 |
| SlIPT2 | aaacgttaacggcgaaaatg | ttcacaatcttggcacttgg | AB690813 |
| SlIPT3 | gcttgctgtcaacgtcaaaa | ttccatgcttgatgtgcttc | JF423320 |
| SlIPT6 | tctcgagagagattgctgctga | gggatcaccagctttaacca | AK324787 |
| SlCKX4 | ggtcagcattggagagtggt | tggtccctccattctctttg | AB690823 |
| SlCKX5 | atcaaatccacgcttttcca | tagccatggatgagggacat | AB690824 |
| ARR1 | ggtcagtggtgaaaaccatg | aactacgctcttttcgcatc | XM\_006345914 |
| ARR4 | ccttgttcgccaacatcacc | gcttaaacgaccccggaagta | XM\_006345914 |
| ARR12 | tcctcagggctatggctc | ctaacttgctagttacaaaaacc | XM\_004251717 |
Additional file 9. Sequences of tomato primers used for qPCR analysis.
